# Strategic enhancement of Cas9 nickase-driven gene amplification

**DOI:** 10.1016/j.crmeth.2026.101544

**Published:** 2026-08-06

**Authors:** Hiroaki Takesue, Satoshi Okada, Takashi Ito

**Affiliations:** 1 Department of Biochemistry, Kyushu University Graduate School of Medical Sciences, Fukuoka 812-8582, Japan

**Keywords:** genome editing, break-induced replication, BITREx, reporter gene, tandem repeat

## Abstract

Gene amplification plays a critical role in evolution and disease and is widely utilized to overexpress valuable gene products in biotechnology. To broaden these applications, we previously developed break-induced replication (BIR)-mediated tandem repeat expansion (BITREx), a method utilizing Cas9 nickase (nCas9) to amplify genetic sequences by driving tandem array expansion through ectopic BIR. Since BITREx efficiency depends on the guide RNA (gRNA) recruiting nCas9 to the array’s flanking regions, here we develop a plasmid-based reporter system in budding yeast for the rapid identification of high-performing gRNAs. Furthermore, we introduce BITREx 2.0, a dual-nicking strategy that targets both sides of the gene array. We demonstrate that BITREx 2.0 is effective for both natural and synthetic arrays, enhancing expansion efficiency by up to an order of magnitude compared to the original single-nicking format. These advancements significantly broaden the applicability and efficiency of nCas9-mediated gene amplification across diverse biological and biotechnological contexts.

## Highlights

• A plasmid reporter predicts gRNA efficiency for BITREx amplification in yeast
• Dual nicking in BITREx 2.0 enables synergistic gene amplification
• BITREx 2.0 performs best with high- and moderate-efficiency gRNAs

## Motivation

Methods for targeted gene amplification have diverse applications in both fundamental and applied biological research. We previously developed a Cas9 nickase-driven method for gene amplification through the expansion of tandem repeats composed of kilobase-sized units. To maximize the practical utility of this method, the selection of guide RNAs (gRNAs) capable of mediating high-efficiency expansion is critical. We thus aimed to facilitate the gRNA selection process by developing a plasmid-based reporter system. Furthermore, we sought to enhance the efficiency of gene amplification by employing a dual-nicking strategy using a specific pair of gRNAs.

## Graphical abstract

**Figure:**
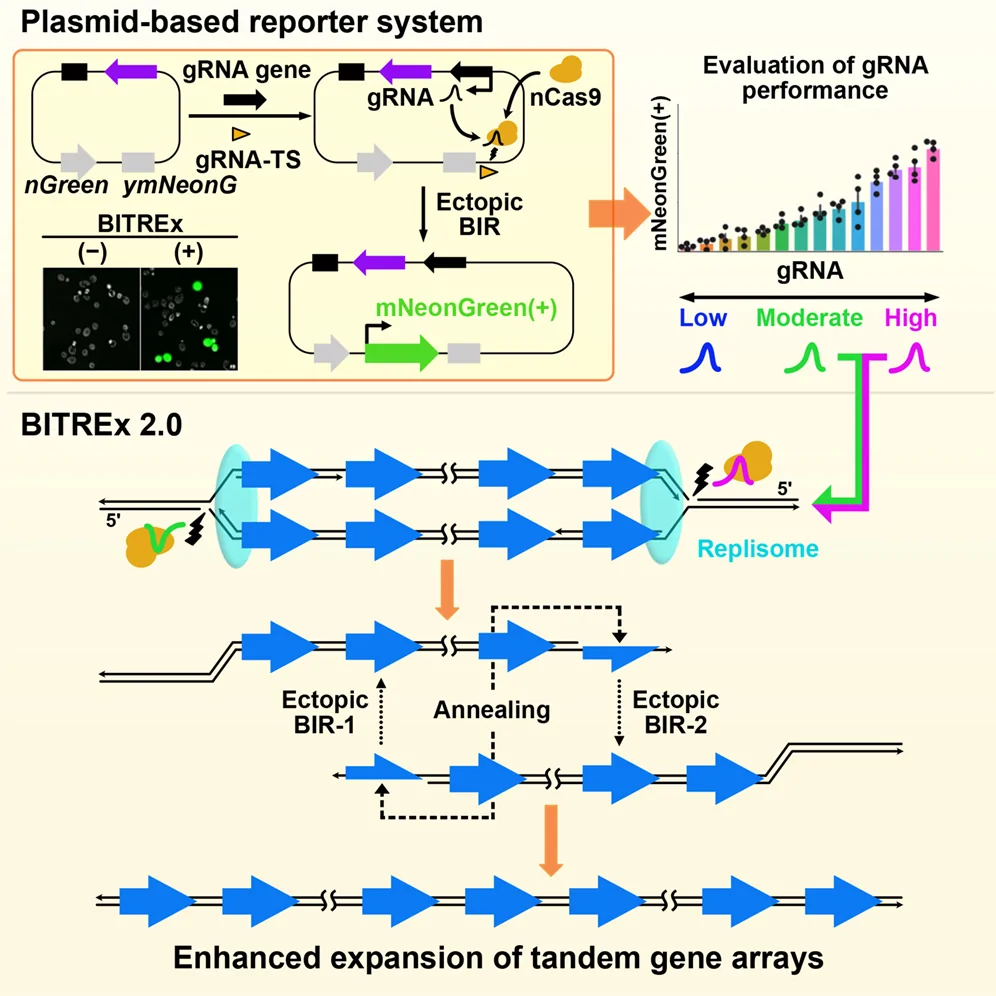

## Teaser

Takesue et al. enhance the nCas9-based gene amplification method BITREx through the development of (1) a plasmid reporter that predicts gRNA performance and (2) BITREx 2.0, a dual-nicking strategy that exploits synergistic interactions between two gRNAs. These advances broaden the utility of BITREx for gene amplification applications.

## Introduction

Gene amplification contributes to adaptive evolution either by increasing the dosage of gene products or by providing the raw genetic material for the generation of functionally diverged paralogs.^1^ A classic example is the mammalian amylase gene family, which consists of three paralogs: *AMY1*, encoding a salivary isozyme, and *AMY2A* and *AMY2B*, encoding pancreatic isozymes. Intriguingly, humans and domestic dogs exhibit elevated copy numbers of *AMY1* and *AMY2A*, respectively. Amylase gene amplification likely represents an adaptation to starch-rich diets introduced by the advent of agriculture, as these amplifications are absent in the respective wild sister lineages, chimpanzees and wolves.^2^^,^^3^

Copy number variations (CNVs) affect not only entire genic segments but also internal genic regions. For instance, the human *LPA* gene encodes apolipoprotein(a), which exhibits a variable number of kringle IV (KIV) domains. This variation arises from the CNV of a ∼5.5-kb segment containing exons 1 and 2 of the *LPA* gene. Crucially, clinical studies have demonstrated an inverse correlation between the number of KIV domains and disease risk, where a lower number of KIV domains are associated with a significantly higher risk for cardiovascular diseases.^4^ Similarly, the contraction of the 3.3-kb D4Z4 macrosatellite repeat results in the derepression of the *DUX4* gene in muscle, thereby inducing facioscapulohumeral muscular dystrophy type 1.^5^ These examples illustrate the involvement of gene-sized tandem repeats in pathogenesis, suggesting that targeted tandem gene array expansion could hold potential for the prevention or treatment of such conditions.

Historically, gene amplification has also been observed in the context of adaptive drug resistance. For instance, cancer cells treated with the folate analog methotrexate (MTX) frequently amplify the dihydrofolate reductase (*DHFR*) gene, which encodes the target protein of MTX, to achieve resistance to this drug.^6^ This MTX-induced *DHFR* amplification has since been repurposed in biotechnology to drive high-level overexpression of recombinant genes of interest, most notably in CHO cells.^7^

Therefore, efficient gene amplification methods would facilitate a wide range of studies in both basic science and biotechnology; however, such tools remain under-developed. Recently, we and others have pioneered a novel class of amplification strategies utilizing Cas9 nickase (nCas9) or prime editor.^8^^,^^9^^,^^10^ We developed paired nicking-induced amplification (PNAmp), which induces the duplication of large genomic segments that are bracketed by direct repeats and have internal replication origins.^8^ By positioning two nCas9 molecules on opposite strands upstream and downstream of these repeats, PNAmp causes sister replication forks to collapse at the nicks, generating a pair of single-ended double-strand breaks (seDSBs). Subsequent end resection exposes the top and bottom strands of the direct repeats as single-stranded DNA (ssDNA), which then anneal to drive the duplication of the target region. We also extended this system using a unique “splint DNA” to facilitate the duplication of regions lacking natural direct repeats. Similar approaches have been reported by two other groups using prime editors to introduce flap sequences for single-strand annealing (SSA).^9^^,^^10^

While these methods are primarily designed for large segmental duplications, we developed break-induced replication (BIR)-mediated tandem repeat expansion (BITREx) for the expansion of tandem gene arrays, or tandem repeats composed of kb-sized units.^11^ In BITREx, a single nCas9 is targeted to the vicinity of an array to introduce a nick on the leading-strand template DNA for the replication fork that traverses the nick-proximal boundary from the interior of the array (Figure 1A). Consequently, a seDSB is generated when the replication fork encounters the nick. While most seDSBs are likely repaired via orthotopic BIR, a subset triggers ectopic BIR: the ssDNA derived from the seDSB invades an upstream unit in the array on the intact sister chromatid to initiate displacement DNA synthesis. This ectopic BIR increases the gene copy number on the broken chromatid while leaving the template chromatid unaltered. Iterative cycles of this process drive rapid array expansion. Indeed, BITREx in the budding yeast *Saccharomyces cerevisiae* has successfully expanded the *CUP1* array—initially comprising 14 copies of the ∼2-kb *CUP1* repeat unit (*CUP1RU*) containing the *CUP1* gene (encoding a copper metallothionein) and the replication origin *ARS810/811*—into Mb-sized arrays containing hundreds to over a thousand units.^11^

**Figure 1:**
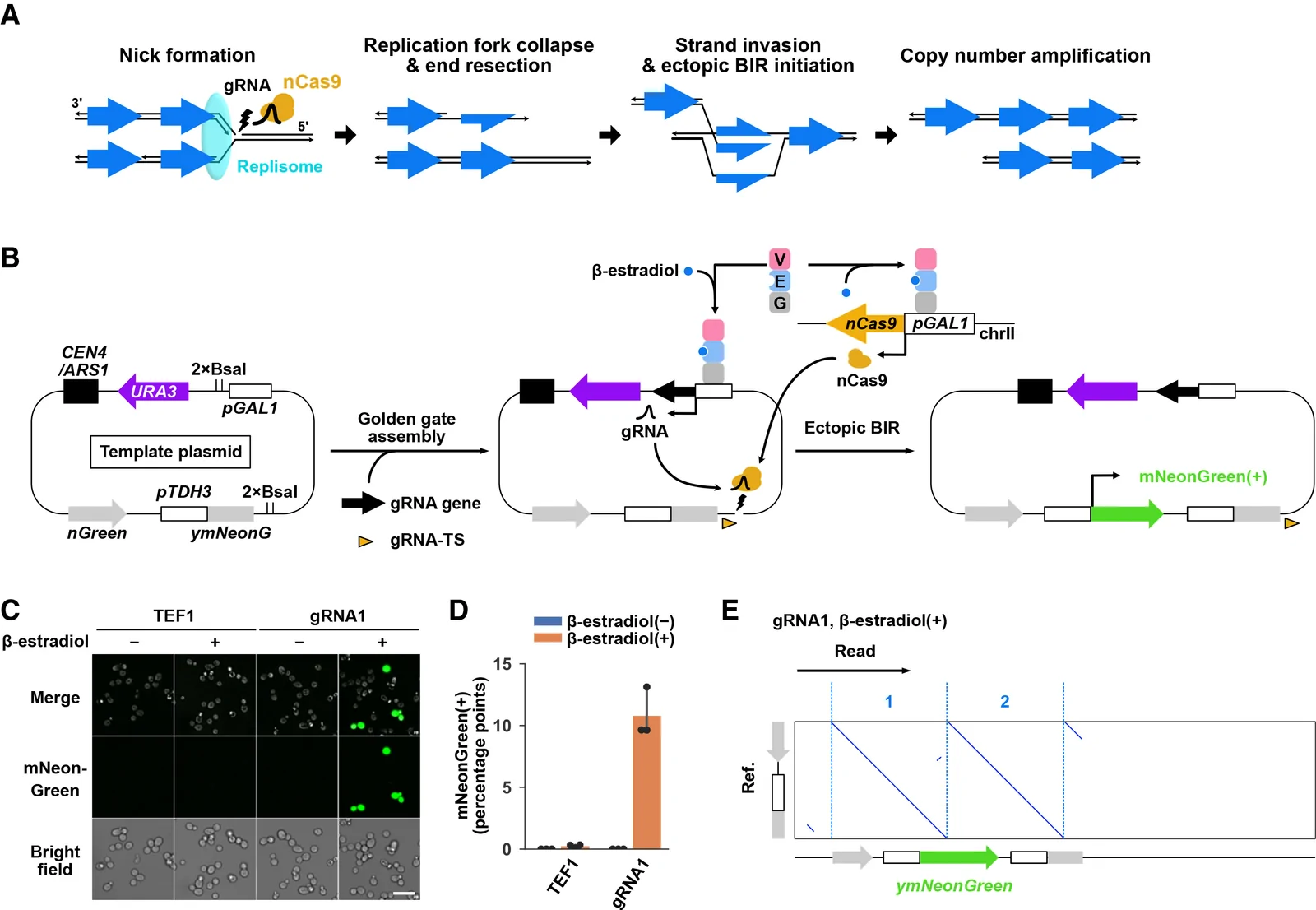
Plasmid-based ectopic BIR reporter system (A) Schematic principle of BITREx. (B) Architecture of the ectopic BIR reporter system. The split-reporter unit is engineered to reconstruct a functional *ymNeonGreen* gene exclusively upon a successful duplication event mediated by ectopic BIR. (C) Fluorescence microscopy of cells undergoing BITREx. Representative images capture the emergence of mNeonGreen signals following induction, indicating successful reporter reconstitution. Scale bars, 20 μm. (D) Efficiency of *ymNeonGreen* gene reconstitution. The graph quantifies the fraction of mNeonGreen-positive cells. TEF1 serves as a non-targeting negative control gRNA. Error bars, standard deviation (SD) (*n* = 3 biological replicates). (E) Genomic validation of reporter unit duplication. A dot plot compares a single nanopore sequencing read against the reference sequence of the split-reporter unit. The two parallel diagonal lines demonstrate the precise, head-to-tail duplication of the unit and the successful reconstruction of an intact *ymNeonGreen* gene.

The efficiency of BITREx is critically dependent on the choice of guide RNA (gRNA). For instance, we identified only six effective gRNAs out of 16 candidates designed in the *CUP1RU* flanking regions, and the underlying factors determining their efficacy remain elusive.^11^ In practice, identifying potent gRNAs is both time-consuming and labor intensive, as it necessitates quantifying copy number alterations via qPCR for each candidate. Furthermore, designing an optimal gRNA in the immediate vicinity of a specific tandem gene array is not always feasible due to sequence constraints. In such instances, it is critical to somehow harness the limited potential of available gRNAs to achieve practically meaningful expansion. Alternatively, if modifications to the flanking sequences are permissible, one may choose to genetically engineer these regions by introducing target sites (TSs) for highly efficient, pre-validated gRNAs.

In this study, we addressed these bottlenecks through two strategic developments. First, to facilitate the rapid screening and selection of high-performing gRNAs, we developed and validated a simple, plasmid-based reporter system utilizing ectopic BIR-mediated reconstruction of a fluorescent protein gene. Second, to fully exploit gRNAs with suboptimal efficiency, we developed a dual-nicking strategy termed BITREx 2.0. Together, these advancements significantly enhance the efficiency, versatility, and applicability of nCas9-driven gene amplification approaches.

## Results

### Development of an ectopic BIR reporter plasmid

BITREx is driven by the iteration of ectopic BIR (Figure 1A). Accordingly, the efficiency of this fundamental process should be a direct, critical determinant of the overall expansion rate. We therefore sought to develop a plasmid-based reporter system capable of quantifying this elementary ectopic BIR event in *S. cerevisiae*.

We designed a centromeric plasmid harboring a gRNA induction unit and a split reporter unit (Figure 1B). The induction unit contains two BsaI sites, facilitating the cloning of gRNA genes under the control of the *GAL1* promoter via Golden Gate Assembly.^12^ The reporter unit utilizes a split version of the *ymNeonGreen* gene, which encodes a yeast codon-optimized variant of the mNeonGreen fluorescent protein. This split configuration consists of an upstream fragment derived from the 3′-portion of *ymNeonGreen* (designated as *nGreen*) and a downstream fragment derived from the 5′-portion (designated as *ymNeonG*). Importantly, these fragments share a 400-bp overlapping sequence (designated as *nG*). A second set of BsaI sites was positioned immediately downstream of the y*mNeonG* fragment to facilitate the insertion of specific gRNA TSs (gRNA-TSs).

The resulting plasmid was transformed into a host strain expressing nCas9 (under the *GAL1* promoter) and the GEV artificial transcription factor (Figure 1B). GEV consists of the yeast Gal4 DNA-binding domain, the human estrogen receptor α ligand-binding domain, and the herpes simplex virus VP16 transactivator domain.^13^ Upon β-estradiol addition, GEV translocates to the nucleus to induce the transcription of both the chromosomal nCas9 gene and the episomal gRNA gene. The gRNA–nCas9 complex is directed to the gRNA-TS on the plasmid to induce a nick; subsequently, a seDSB is generated when a replication fork, initiated from the replication origin *ARS1*, migrates through the *nGreen* and *ymNeonG* fragments to encounter the nick, thereby triggering BIR. We hypothesized that a fraction of the ssDNA generated from the seDSB would invade the upstream *nGreen* fragment via the shared *nG* homology, initiating ectopic BIR to reconstitute a functional *ymNeonGreen* gene under the constitutive *TDH3* promoter.

Indeed, yeast cells exhibited robust fluorescence only when they harbored both the specific gRNA and its corresponding TS and were grown in the presence of β-estradiol (Figures 1C and 1D). Furthermore, nanopore sequencing of the recovered plasmid DNA, followed by dot plot analysis against the reference sequence of the split *ymNeonGreen* reporter unit, confirmed the anticipated tandem duplication and gene reconstitution (Figure 1E). These results provide direct molecular evidence validating the concept of our reporter system.

### Prediction of gRNA performance in BITREx by the ectopic BIR reporter plasmid

We next evaluated whether the episomal reporter activities for individual gRNAs could predict their performance at chromosomal loci. For this purpose, we selected 13 gRNA-TSs from the terminal regions of the bacteriophage λ genome (Figure 2A) and established yeast strains where the original gRNA1-TS flanking the *CUP1* array was individually replaced by each λ-derived TS using Cas12a-mediated genome editing (Figure 2B). Concurrently, we generated a series of reporter plasmids carrying both these TSs and their corresponding gRNAs and measured their activities based on the increase in mNeonGreen-positive cells following β-estradiol treatment (Figure 2C). After inducing BITREx for 3 days, we quantified the population-average *CUP1* copy numbers by qPCR and estimated the number of cell generations based on culture absorbance (OD_620_). By integrating these measurements, we calculated the copy number alterations per generation (CNA/G) as a standardized metric for BITREx efficiency (see STAR Methods; Figure 2D). A strong correlation was observed between the reporter activity and the CNA/G value (R^2^ = 0.803), demonstrating that the reporter system accurately predicts gRNA performance at genomic targets (Figure 2E). In stark contrast, three widely used *in silico* programs^14^^,^^15^^,^^16^ failed to predict the efficiency of these gRNAs in BITREx (Figure 2F).

**Figure 2:**
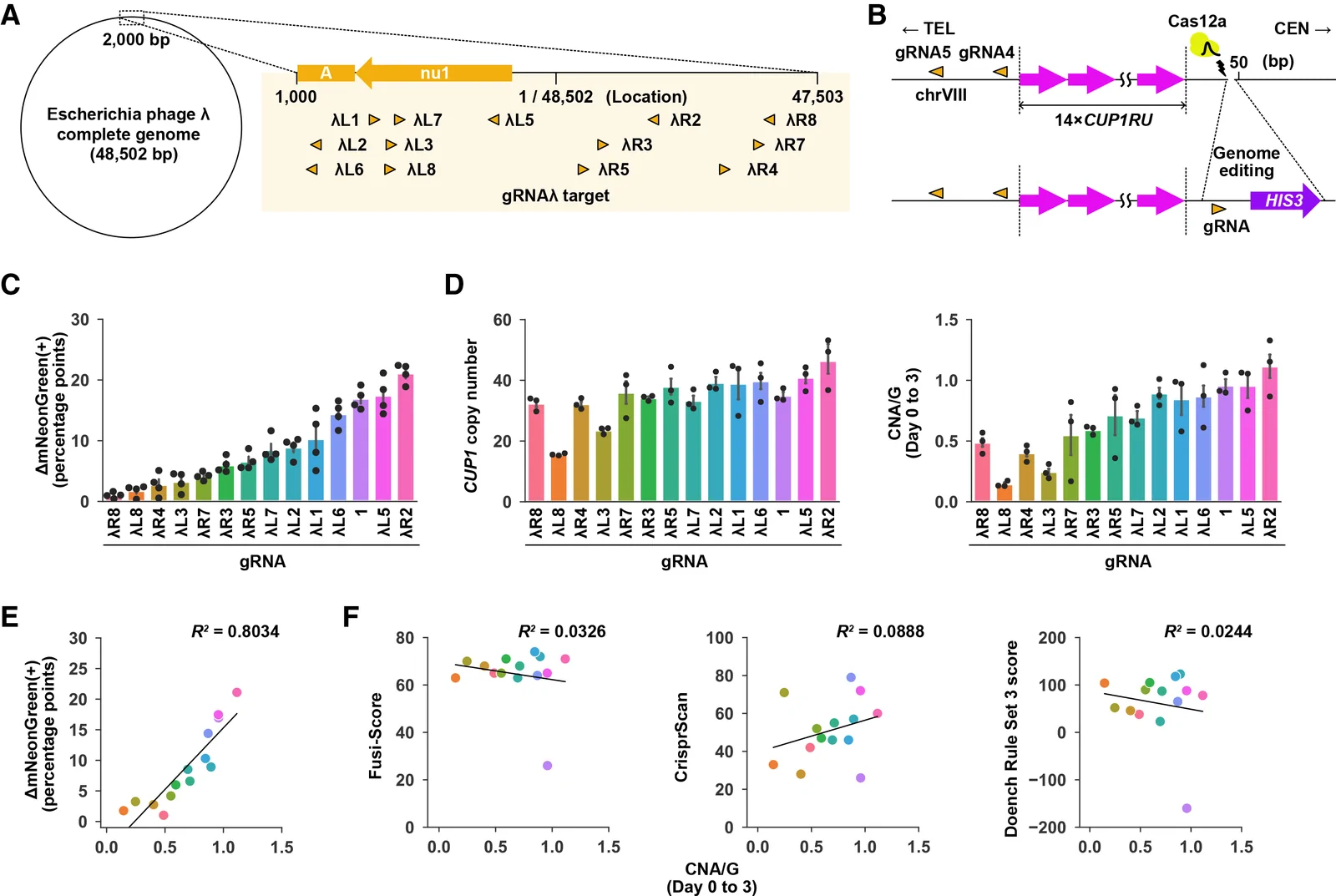
Validation of the ectopic BIR reporter system for predicting gRNA performance (A) Selection of 13 candidate gRNAs targeting the λ phage genome sequence. (B) Cas12a-mediated *CUP1* locus engineering. The original gRNA1-TS is replaced with specific λ-derived gRNA-TSs to facilitate standardized testing. (C) Evaluation of reporter activities on episomal target sites. Efficiency is quantified as ΔmNeonGreen(+) (percentage points), the increased frequency of mNeonGreen-positive cells after 24 h of BITREx induction with β-estradiol, compared with the β-estradiol(−) condition. Error bar, SD (*n* = 4 biological replicates). (D) Performance of λ-derived gRNAs at chromosomal loci. (Left) *CUP1* copy number at day 3; (right) CNA/G. Error bar, SD (*n* = 3 biological replicates). (E) Correlation between episomal and chromosomal performance. Linear regression shows the relationship between reporter activities (ΔmNeonGreen(+)) and CNA/G values of *CUP1* array expansion. (F) Predictive power of computational gRNA design tools. For each gRNA, Doench/Fusi score (Rule Set 2 on-target score),^14^ CRISPRscan score,^15^ and Doench Rule Set 3 score^16^ are plotted against its CNA/G value.

We further assessed whether the reporter system could identify efficient gRNAs within natural genomic contexts. We tested a series of gRNAs targeting the regions flanking the *CUP1* array (Figure S1A), which had previously shown varying efficiencies in BITREx.^11^ Although the distance between the nick and the downstream *nG* fragment remained constant in the reporter plasmids (41 bp), the corresponding distances from the nick to the *CUP1RU* in the genome varied significantly among these gRNAs (41–471 bp). Despite these differences, the reporter activities showed a good correlation with CNA/G values (R^2^ = 0.709), confirming that the system can effectively distinguish high-performing gRNAs from low-performing ones in a natural genomic setting (Figures S1B–S1E).

Together, these results highlight the utility of our reporter system for selecting optimal gRNAs or, at least, filtering out ineffective candidates. Interestingly, we found that even gRNAs identified as “low performing” can contribute to gene amplification in a unique manner, as described in the later sections.

### Rationale for BITREx 2.0, a dual-nicking strategy for improved tandem array expansion

Although the reporter system described above facilitates the identification of high-efficiency gRNAs, their design can still be restricted by the sequence constraints of specific genomic loci. In practice, researchers often encounter genomic targets where suboptimal sequence composition does not allow the designing of high-efficiency gRNAs. In such instances, it is critical to maximize the potential of these available gRNAs to achieve practically meaningful expansion.

To this end, we conceived BITREx 2.0, a dual-nicking strategy that simultaneously utilizes two gRNAs (Figure 3A). Consider a tandem gene array consisting of *N* repeat units, each harboring the target gene and an internal replication origin. In this configuration, replication forks are expected to migrate outward from within the array toward both flanking regions. Consequently, BITREx initiation could, in principle, occur at either or even both ends of the array. When ectopic BIR events are triggered at both flanks, an additive effect is anticipated, resulting in extended arrays in both daughter cells. While a seDSB may be repaired via orthotopic BIR (restoring the original array length), expansion is achieved as long as at least one seDSB undergoes ectopic BIR (Figure 3A).

**Figure 3:**
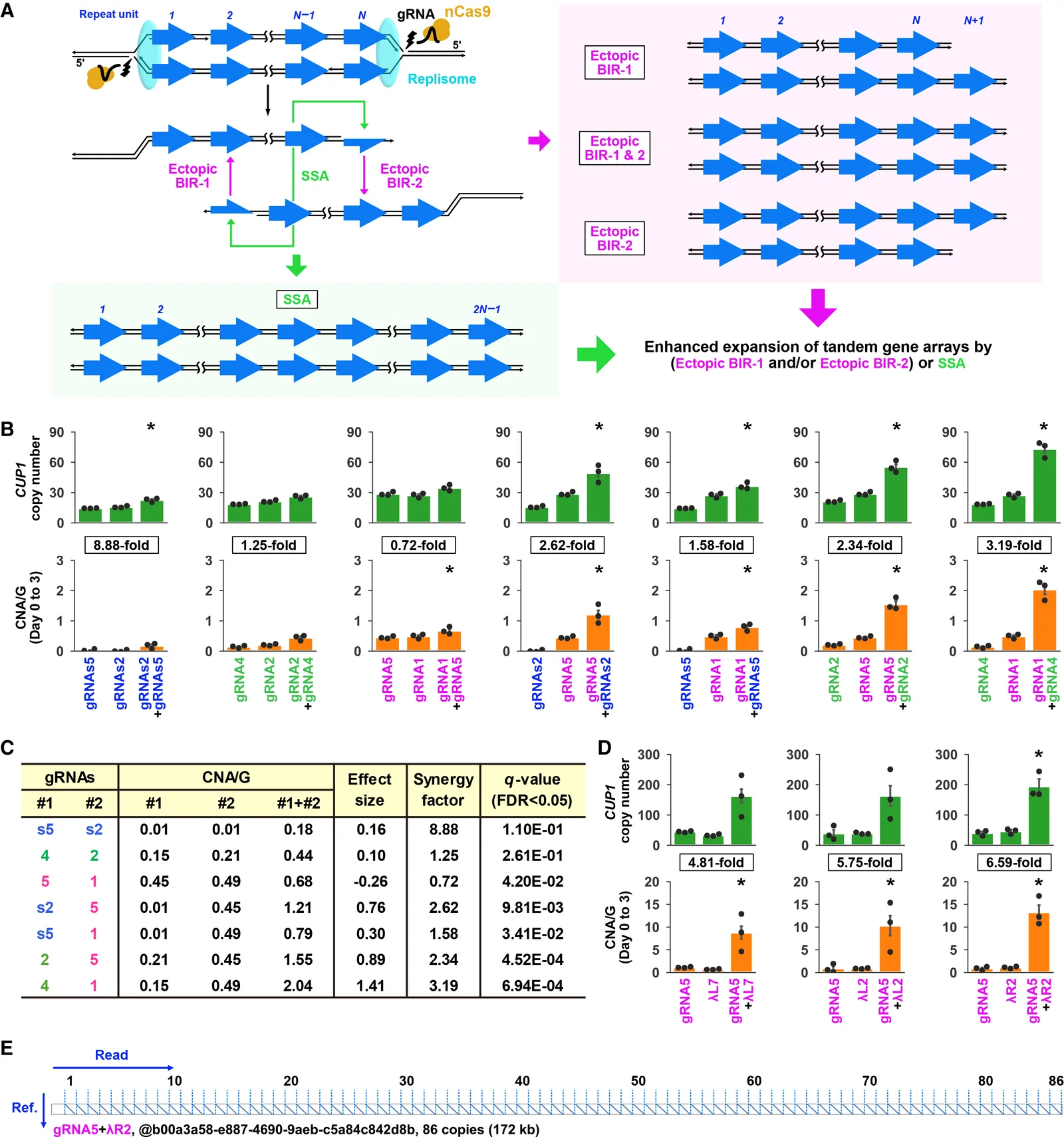
BITREx 2.0-mediated acceleration of *CUP1* array expansion (A) Rationale for BITREx 2.0. Dual-nicking at both flanks of a tandem gene array can trigger ectopic BIR from either or both ends (BIR-1 and BIR-2). For illustrative simplicity, each BIR event is depicted as adding a single repeat unit to the broken sister chromatid. Alternatively, SSA between distal repeat units (*1*st and *N*th) results in an expansion increasing the unit copy number from *N* to *2N−1*. (B) *CUP1* array expansion via dual nicking. Expansion efficiency is evaluated across seven gRNA combinations: three pairs with similar efficiencies and four pairings of high-performing gRNAs with moderate or low-performing ones. gRNA names are color-coded by performance: magenta (high), green (moderate), and blue (low). (Top) *CUP1* copy number at day 3; (bottom) CNA/G values, with synergy factors indicated in the boxes. Error bar, SD (*n* = 3 biological replicates). ∗*q* < 0.05 (Benjamini–Hochberg adjusted). (C) Summary table for the data shown in (B). Individual gRNAs are listed in ascending order of their single-nicking BITREx efficiencies (columns #1 to #2) and color-coded as in (B). (D) Representative BITREx 2.0 performance using synthetic λ-derived TSs. (Top) *CUP1* copy number at day 3; (bottom) CNA/G values, with synergy factors indicated in the boxes. Error bar, SD (*n* = 3 biological replicates). ∗*q* < 0.05 (Benjamini–Hochberg adjusted). (E) Structural validation by nanopore sequencing. A dot plot of a representative long read from cells treated with gRNA5 and gRNAλR2 indicates the presence of *CUP1* array composed of 86 repeat units. For the distribution of estimated *CUP1* copy numbers, see Figure S2C.

Notably, end resection of the paired seDSBs could expose the top strand of the first repeat unit and the bottom strand of the *N*th repeat unit as ssDNA (Figure 3A). Annealing between these ssDNAs would drive the duplication of the entire array, increasing the repeat number from *N* to *2N−1*. Subsequent replication, driven by replisomes migrating from both sides of the array, would produce two daughter cells each carrying *2N−1* repeat units (Figure 3A). This proposed mechanism is analogous to PNAmp, which we previously reported for the duplication of large genomic regions flanked by direct repeats.^8^

We thus hypothesized that the simultaneous targeting of both flanking sites—BITREx 2.0—would exploit these possible pathways to drive array expansion more efficiently than the conventional single-nicking BITREx.

### Application of BITREx 2.0 to the *CUP1* array

As a model to evaluate the feasibility of BITREx 2.0, we targeted the natural *CUP1* array, composed of 14 repeat units, using three gRNAs from each of its flanks (see Figure S1A for their genomic positions). Among these, gRNA1 and gRNA5 were pre-validated as high efficiency, gRNA2 and gRNA4 as moderate efficiency, and gRNAs2 and gRNAs5 as low efficiency, respectively (Figure S1C; for the standardized color-coding of these gRNA names, refer to the Figure 3B legend).

To simulate a “worst-case scenario” where only low-efficiency gRNAs are available, we combined gRNAs5 and gRNAs2, each of which individually yielded a CNA/G value of only 0.01 (Figures 3B and 3C). Remarkably, the gRNAs5+gRNAs2 combination yielded an absolute CNA/G value of 0.18—a performance comparable to that of single, modestly effective gRNAs like gRNA2 (0.21) or gRNA4 (0.15) (Figures 3B and 3C). Similarly, combining two moderate-efficiency gRNAs (gRNA4+gRNA2) yielded a CNA/G value of 0.44, approaching the performance of high-efficiency gRNAs (Figures 3B and 3C). Here, we introduce the “synergy factor”—defined as the observed dual-gRNA CNA/G divided by the sum of their individual baseline efficiencies—as a descriptive metric to evaluate combinations (see STAR Methods). We also calculated the synergy factor using absolute copy number values (Figure S2A). Crucially, to maintain statistical rigor, we defined an effect as formally synergistic only when a two-way ANOVA revealed a statistically significant interaction between the two gRNAs after Benjamini–Hochberg correction for multiple testing (*q* < 0.05). While the two combinations described above yielded CNA/G values exceeding their respective expected additive effects, two-way ANOVA indicated that their interactions did not reach statistical significance (Figure 3C). Intriguingly, however, the combination of two high-efficiency gRNAs (gRNA1+gRNA5) resulted in a CNA/G value of 0.68. While this surpassed the values for gRNA1 (0.49) or gRNA5 (0.45) alone, it fell short of the expected additive effect, indicating a significant negative interaction (0.72-fold, *q* = 4.20 × 10^−2^) (Figures 3B and 3C).

Since the absolute CNA/G value ultimately dictates the practical utility, we next investigated whether BITREx 2.0 could further elevate the performance of high-efficiency gRNAs (i.e., gRNA1 and gRNA5) beyond the level achieved by their own combination (0.68). Intriguingly, this threshold was surpassed when a high-efficiency gRNA was paired with a moderate- or even low-efficiency counterpart. For instance, the gRNA1+gRNA4 and gRNA5+gRNA2 combinations yielded CNA/G values of 2.04 and 1.55, which not only significantly exceed the 0.68 achieved by gRNA1+gRNA5 but also represent 3.19-fold and 2.34-fold increases over their respective expected additive effects (*q* = 6.94 × 10^−4^ and 4.52 × 10^−4^) (Figures 3B and 3C). Even when high-efficiency gRNAs were paired with low-efficiency candidates (i.e., gRNA1+gRNAs5 and gRNA5+gRNAs2, each partner having an individual efficiency of only 0.01), the resulting CNA/G values reached 0.79 and 1.21, respectively. Both combinations outperformed the gRNA1+gRNA5 pair, exhibiting synergistic enhancements of 1.58- and 2.62-fold over their additive expectations (0.50 and 0.46) (*q* = 3.41 × 10^−2^ and 9.81 × 10^−3^) (Figures 3B and 3C).

To further explore the versatility and potential of BITREx 2.0, we applied it to modified *CUP1* arrays in which the native gRNA1-TS was replaced with various λ-derived TSs (Figure 2B). Evaluation of these strains revealed that individual gRNA performances were noticeably modulated by the local genomic environment, including the insertion of the *HIS3* marker and the λ-derived TS (see Figure S2B for details). Nevertheless, BITREx 2.0 using λ-derived gRNAs paired with either gRNA4 or gRNA5 demonstrated significant synergistic effects across multiple combinations. Specifically, two-way ANOVA of CNA/G values identified statistically significant interactions for 8 and 11 out of the 13 λ-derived gRNAs when combined with gRNA4 and gRNA5, respectively, including negative interactions (Figure S2B). Several combinations with gRNA5 yielded remarkable results, driving *CUP1* array expansion from 14 to over 100 units (e.g., λR2, λL2, and λL7) (Figure 3D). Notably, two of these combinations achieved CNA/G values exceeding 10 (λR2 and λL2), underscoring the high-capacity amplification potential of the BITREx 2.0 system (Figure 3D).

To verify these findings, we performed nanopore sequencing of genomic DNA isolated from these strains. The results confirmed that the *CUP1* array expansion occurred *in situ*, as exemplified by a dot plot analysis of long reads against the *CUP1RU* reference sequence (Figures 3E and S2C). Additional nanopore read analysis revealed no detectable levels of translocation involving the *CUP1* locus nor any evidence of aneuploidy affecting chromosome VIII, consistent with our previous observations for the conventional BITREx system^11^ (Figures S2D and S2E).

Collectively, these findings underscore that low- or moderate-efficiency gRNAs are not merely suboptimal tools; rather, they can serve as essential partners that significantly drive tandem gene array expansion within the BITREx 2.0 framework. Therefore, BITREx 2.0 represents a powerful strategy not only for harnessing the latent potential of marginal gRNAs but also for further augmenting the expansion capacity of high-performance gRNAs, achieving an enhancement that can exceed the original BITREx by an order of magnitude.

### Complex mechanisms inferred from BITREx 2.0 in mutant cells

To dissect the molecular mechanisms underlying BITREx 2.0, we systematically examined the roles of key genes involved in the BIR and SSA pathways. In the BIR pathway, Rad51 is essential for the initial strand invasion into homologous sequences, while Pol32 (a non-essential subunit of DNA polymerase δ) and the Pif1 DNA helicase are required to drive extensive displacement synthesis.^17^ Conversely, Rtt109—the sole H3K56 acetyltransferase—acts as a negative regulator of BIR.^18^ While H3K56 acetylation deposited during S phase appears to promote canonical pathways following a replication fork collapse, the loss of Rtt109 likely triggers a mechanistic shift toward BIR-mediated rolling-circle amplification, ultimately leading to rDNA hyperamplification.^19^ In the SSA pathway, Rad52 facilitates the annealing of complementary sequences, whereas the Rad1–Rad10 complex, alongside Msh2 and Msh3, is required for the subsequent cleavage of non-homologous 3′ flaps.^17^ Hence, if BIR is the predominant mechanism in BITREx 2.0, its efficiency should be suppressed in *rad51*Δ, *pol32*Δ, and *pif1-m2* cells and derepressed in *rtt109*Δ cells, as observed for the single-nicking BITREx.^11^ Conversely, if SSA is the predominant mechanism in BITREx 2.0, its efficiency should be suppressed in *rad52*Δ, *rad1*Δ, *msh2*Δ, and *msh3*Δ cells but not in *rad51*Δ cells, as observed for PNAmp.^8^

We generated mutant profiles for individual gRNAs/gRNA pairs in terms of CNA/G and CNA values in mutant strains (Figures 4A and 4B). It should be noted that, because these mutants showed slower growth than the wild-type strain (Figure S3A), the increase in CNA/G values was driven not only by an increase in CNA but also by a decrease in G. Accordingly, the fold changes in CNA were less pronounced than those in CNA/G (see Figure S3B for details). In our previous study, we utilized gRNA1 and gRNA6 to demonstrate that the original single-nicking BITREx was suppressed in *rad51*Δ, *pol32*Δ, and *pif1-m2* cells, while being derepressed in *rtt109*Δ cells.^11^ This pattern was observed for gRNA2, gRNA4, and gRNA5 in the present study, further confirming that conventional BITREx is primarily mediated by canonical BIR (Figure 4A).

**Figure 4:**
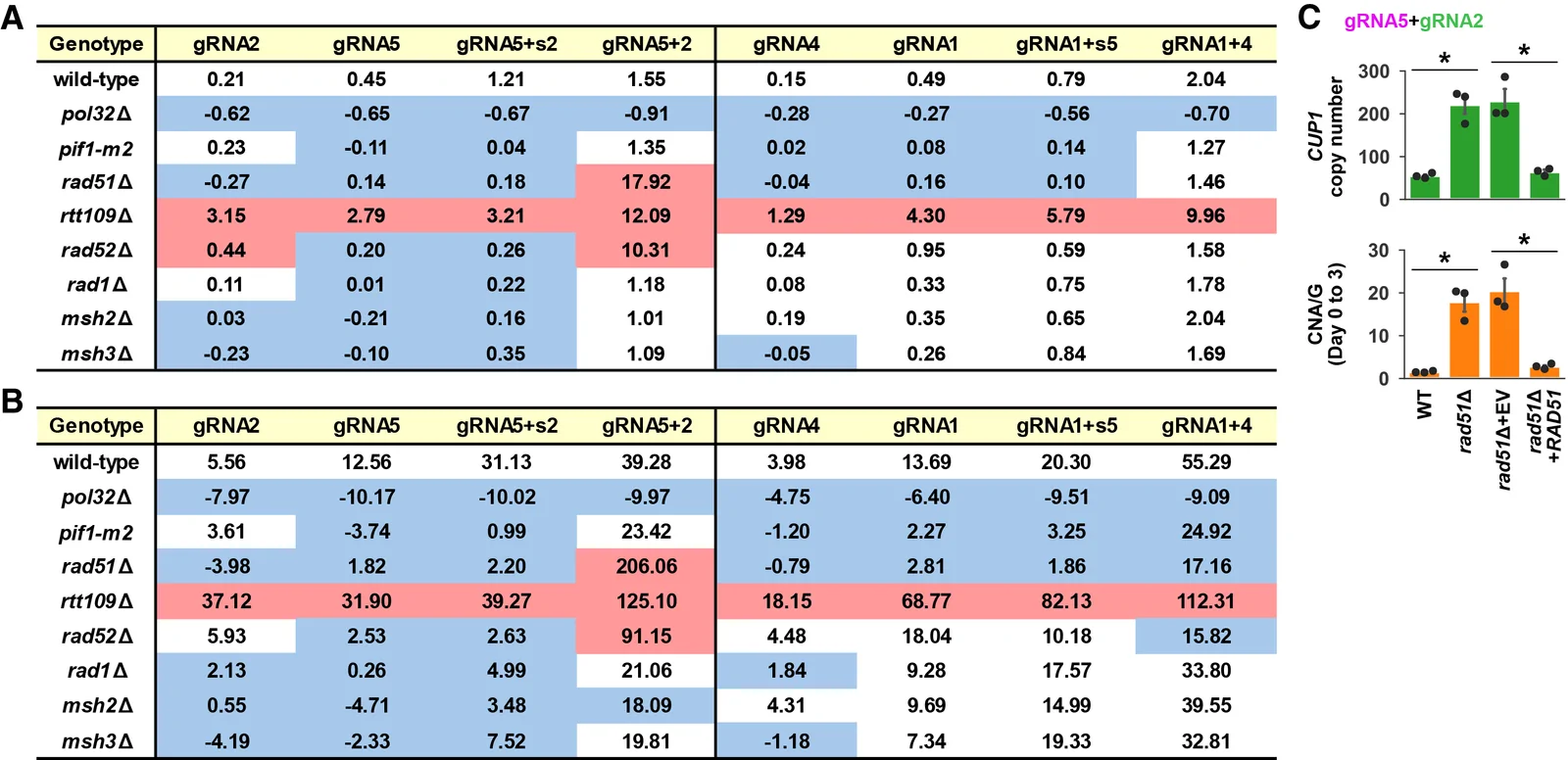
Genetic dissection of the BITREx 2.0 mechanisms (A) Genetic profiles of BITREx 2.0 in mutant strains defective in canonical BIR and SSA. The summary table displays the average CNA/G values for the indicated gRNAs and gRNA pairs across various mutant backgrounds (*n* = 3 biological replicates). To highlight significant genetic dependencies, gRNA-mutant combinations exhibiting a >2-fold decrease or increase compared to the corresponding gRNA-wild-type combinations are heatmapped in light blue and light red, respectively. (B) Effects of deleting genes involved in canonical BIR and SSA. Data are presented similarly to (A) but summarized for CNA from day 0 to day 3 instead of CNA/G. (C) Functional validation of Rad51-mediated suppression of BITREx 2.0 with the gRNA5+gRNA2 combination. *CUP1* array expansion is examined in the wild-type cells, *rad51*Δ cells, and *rad51*Δ cells harboring either an empty control vector or a *RAD51*-expression plasmid. (Top) *CUP1* copy number at day 3; (bottom) CNA/G. Error bar, SD (*n* = 3 biological replicates). ∗*p* < 0.05 (Student’s *t* test).

In contrast, BITREx 2.0 exhibited a divergent dependency on BIR-related genes depending on the gRNA configuration. Combinations of high- and low-efficiency gRNAs (e.g., gRNA1+gRNAs5 and gRNA5+gRNAs2) displayed mutant profiles reminiscent of canonical BIR (Figure 4A). However, the gRNA1+gRNA4 combination showed a diminished requirement for *RAD51* and *PIF1* (Figure 4A). Most strikingly, BITREx 2.0 efficiency using the gRNA5+gRNA2 pair was enhanced by over an order of magnitude in *rad51*Δ cells (Figure 4A). We confirmed that episomal expression of *RAD51* successfully rescued this phenotype, suppressing the hyper-expansion observed in *rad51*Δ cells (Figure 4C). To further explore this finding, we constructed a strain in which *RAD51* expression is driven by a doxycycline (Dox)-inducible Tet-On promoter (*P7tet.1*)^20^ (Figure S3C). While the result of the inducible-expression experiment should be considered preliminary due to the modest magnitude of change and experimental variability, it should be regarded as corroborative evidence, with the primary conclusion being supported by the rescue experiment (Figure S3D). These results collectively suggested that Rad51 is not essential for, and may potentially limit, BITREx 2.0-mediated expansion—a notable contrast to its essential role in single-nicking BITREx.

Regarding SSA-related genes, the deletion of factors other than *RAD52* had only marginal effects on BITREx 2.0 mediated by either the gRNA1+gRNA4 or gRNA5+gRNA2 combinations (Figure 4A). This observation stands in stark contrast to our previous findings with PNAmp, where these SSA components were essential for efficient duplication.^8^ Remarkably, the gRNA5+gRNA2 pair exhibited significantly higher expansion efficiency in *rad52*Δ cells compared to wild-type cells.

Together, these data indicate that BITREx 2.0 is mediated neither by canonical BIR nor by simple SSA in isolation. Instead, our findings suggest that both pathways contribute in a variably composite manner, with their relative involvement dictated by the specific combination of gRNAs employed. Furthermore, the unexpectedly robust synergistic enhancements observed with the gRNA5+gRNA2 pair in *rad51*Δ and *rad52*Δ cells underscore the complex, multi-layered nature of the mechanisms driving BITREx 2.0, likely involving alternative or non-canonical ones.

### Application of BITREx 2.0 to synthetic interrupted two-unit arrays

We have previously shown that BITREx is applicable to two-unit arrays separated by an intervening sequence.^11^ Such a structure is readily generated via plasmid integration. For instance, when an integrative plasmid carrying a single *CUP1RU* is transformed into a strain harboring a single *CUP1RU*, a recombinant strain is obtained that carries the plasmid sequence bracketed by two *CUP1RUs* at the *CUP1* locus. If the plasmid contains an exogenous gene of interest (GOI), its copy number can be subsequently increased through BITREx. Since the GOI is between the flanking repeat units, it will be amplified to a copy number exactly one less than that of the repeat units. Given that this strategy serves as a versatile platform for the amplification of diverse exogenous GOIs, we sought to apply BITREx 2.0 to such synthetic, interrupted two-unit arrays to evaluate its performance in a heterologous context.

We generated an interrupted two-unit *CUP1RU* array containing a 5.1-kb intervening sequence that carries the *pNOP1-ymNeonGreen* expression cassette, *ARS305*, and the *KanMX* marker (Figure 5A) in the Dox-inducible *RAD51* strain (Figure S3C). We observed a consistent greater-than-additive effect with the gRNA5+gRNA2 combination, regardless of whether *RAD51* expression was induced by Dox (Figures 5B and S4A). Intriguingly, BITREx 2.0 using the gRNA1+gRNA4 combination showed a pronounced enhancement specifically in the absence of Dox. Nanopore sequencing confirmed the faithful amplification of the intervening sequence within the array (Figures 5C and S4B). Correspondingly, the increase in copy number led to a concomitant enhancement of mNeonGreen fluorescence, thereby validating a clear gene-dosage effect (Figures 5D and S4C). These results collectively demonstrate that BITREx 2.0 is highly effective for the overexpression of exogenous GOIs embedded in the intervening sequence of an interrupted two-unit array. Furthermore, they are consistent with the possibility that *RAD51* may compromise the efficiency of BITREx 2.0 in some cases (Figures 4B and S3D).

**Figure 5:**
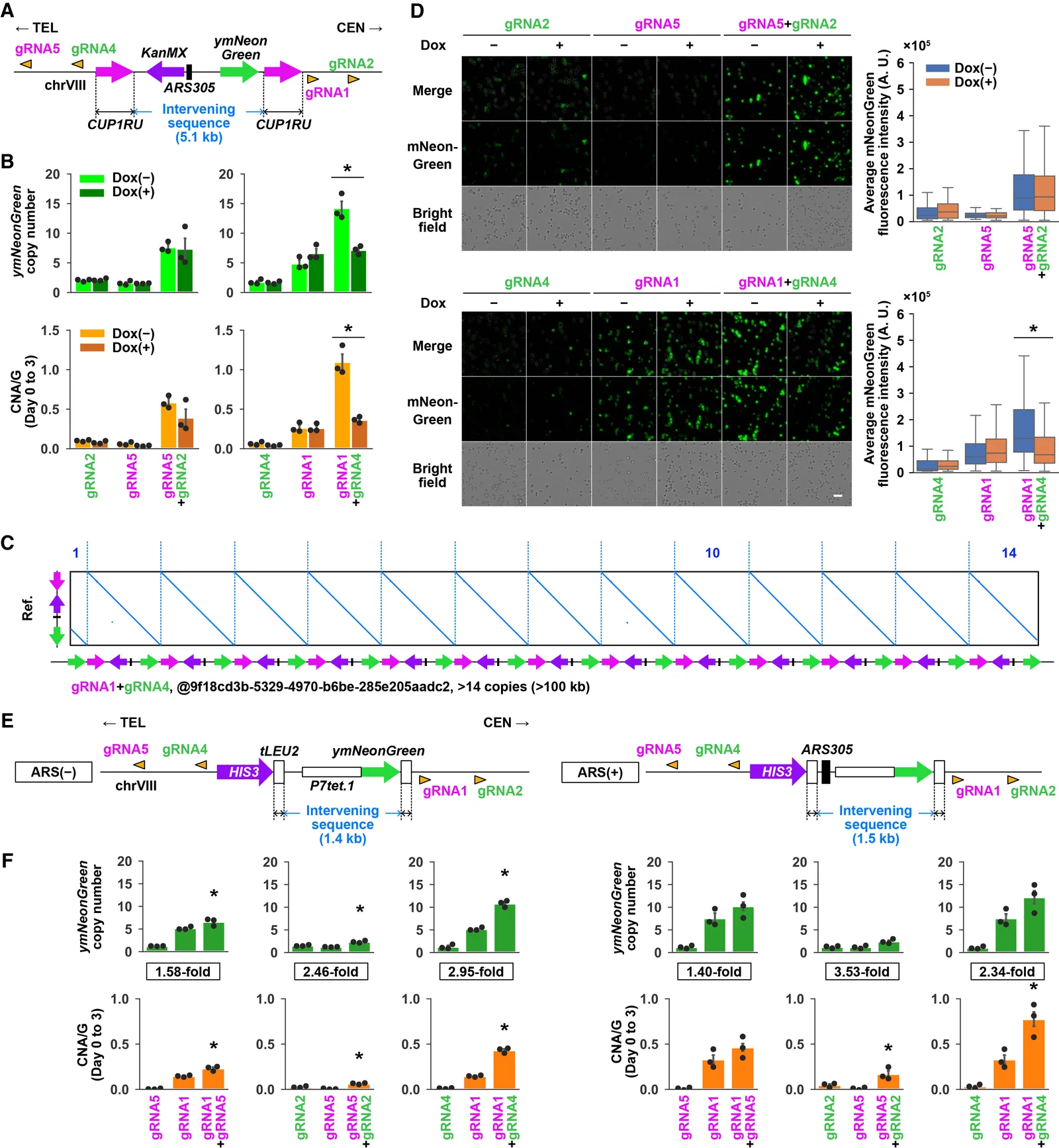
BITREx 2.0-mediated acceleration of interrupted two-unit array expansion (A) Architecture of an interrupted two-unit *CUP1* array. The 5.1-kb intervening sequence carries the *pNOP1-ymNeonGreen* gene,^11^*ARS305*, and the *KanMX* marker. This array is constructed in the strain for Dox-dependent expression of *RAD51* (Figure S3C). (B) Expansion of the interrupted two-unit *CUP1* array by BITREx 2.0. Two gRNA pairs are used to drive expansion in the presence and absence of Dox. (Top) *ymNeonGreen* copy number at day 3; (bottom) CNA/G. Error bar, SD (*n* = 3 biological replicates). ∗*p* < 0.05 (Student’s *t* test). (C) Structural validation by nanopore sequencing. A dot plot of a representative long read reveals an amplification of the intervening sequence from one to ≥14 copies. For the distribution of estimated *CUP1* copy numbers, see Figure S4B. (D) Fluorescence microscopy of cells undergoing BITREx. Representative images are shown in the left. The boxplots in the right indicate the distribution of the average mNeonGreen fluorescence intensity. The bottom and top of the box indicate the first and third quantiles, respectively. The bar in each box indicates the median value, and the error bars represent the range of values. ∗*p* < 0.001 (Mann–Whitney U test). Scale bars, 20 μm. (E) Architecture of interrupted two-unit *tLEU2* arrays. The intervening sequence carries a Dox-inducible *ymNeonGreen* expression cassette with and without *ARS305*. Note that *HIS3* insertion increased the distance between the array and target sites of gRNA4 and gRNA5. (F) Expansion of interrupted two-unit *tLEU2* arrays. Three gRNA pairs are used. (Top) *ymNeonGreen* copy number at day 3; (bottom) CNA/G values, with synergy factors indicated in the boxes. Error bar, SD (*n* = 3 biological replicates). ∗*q* < 0.05 (Benjamini–Hochberg adjusted).

We applied BITREx 2.0 to an alternative interrupted two-unit array, where two copies of the *LEU2* terminator (*tLEU2*) bracket a Dox-inducible *crtI* gene (Figure S4D). This gene, derived from the red yeast *Xanthophyllomyces dendrorhous*, encodes a key enzyme in β-carotene biosynthesis. The entire cassette, along with its flanking gRNA-TSs, was integrated into a genomic safe-harbor site on chromosome XI.^21^ Two configurations were compared: one with asymmetrical flanking TSs (gRNA5-TS and gRNAλR2-TS) and the other with symmetrical TSs (gRNAλR2-TS at both flanks) (Figure S4D). In the gRNA5+gRNAλR2 configuration, BITREx 2.0 successfully mediated array expansion (Figures S4E and S4F). Notably, while gRNA5 failed to initiate expansion in a single-nicking format—possibly because the insertion of the *HphMX* selection marker increased the distance between the nick and the array boundary—it functioned effectively as a synergistic partner to gRNAλR2. Conversely, the gRNAλR2+gRNAλR2 combination failed to expand the array (Figures S4E and S4F). This observation parallels the suboptimal expansion efficiency previously noted in BITREx 2.0 when utilizing a pair of high-efficiency gRNAs (Figures 3B and 3C).

We also investigated the role of internal replication origins using interrupted two-unit arrays, in which two copies of *tLEU2* bracket a Dox-inducible *ymNeonGreen* gene with or without an adjacent *ARS305* (Figure 5E). In our original BITREx 2.0 model, an internal replication origin was hypothesized to be essential, as it provides the replication forks that migrate outward toward both flanking regions to trigger BITREx or PNAmp (Figure 3A). Unexpectedly, we observed a synergistic effect of BITREx 2.0 even on the ARS-less array, although the expansion efficiency was reduced compared to the ARS-bearing counterpart (Figure 5F). Although it is formally possible that the *P7tet.1-ymNeonGreen* construct unexpectedly serves as a cryptic replication origin, this observation further suggests that BITREx 2.0 can be mediated by alternative mechanisms beyond the originally hypothesized scheme.

### Expansion of an expansion-refractory array by BITREx 2.0

We hypothesized that BITREx 2.0 would be particularly advantageous for gene arrays that exhibit limited expansion efficiencies under the original single-nicking scheme. To test this, we applied BITREx 2.0 to an uninterrupted two-unit *CUP1* array (Figure 6A); we previously identified this minimal substrate as being significantly more resistant to expansion than the natural 14-unit array during the initial 3 days of induction.^11^

**Figure 6:**
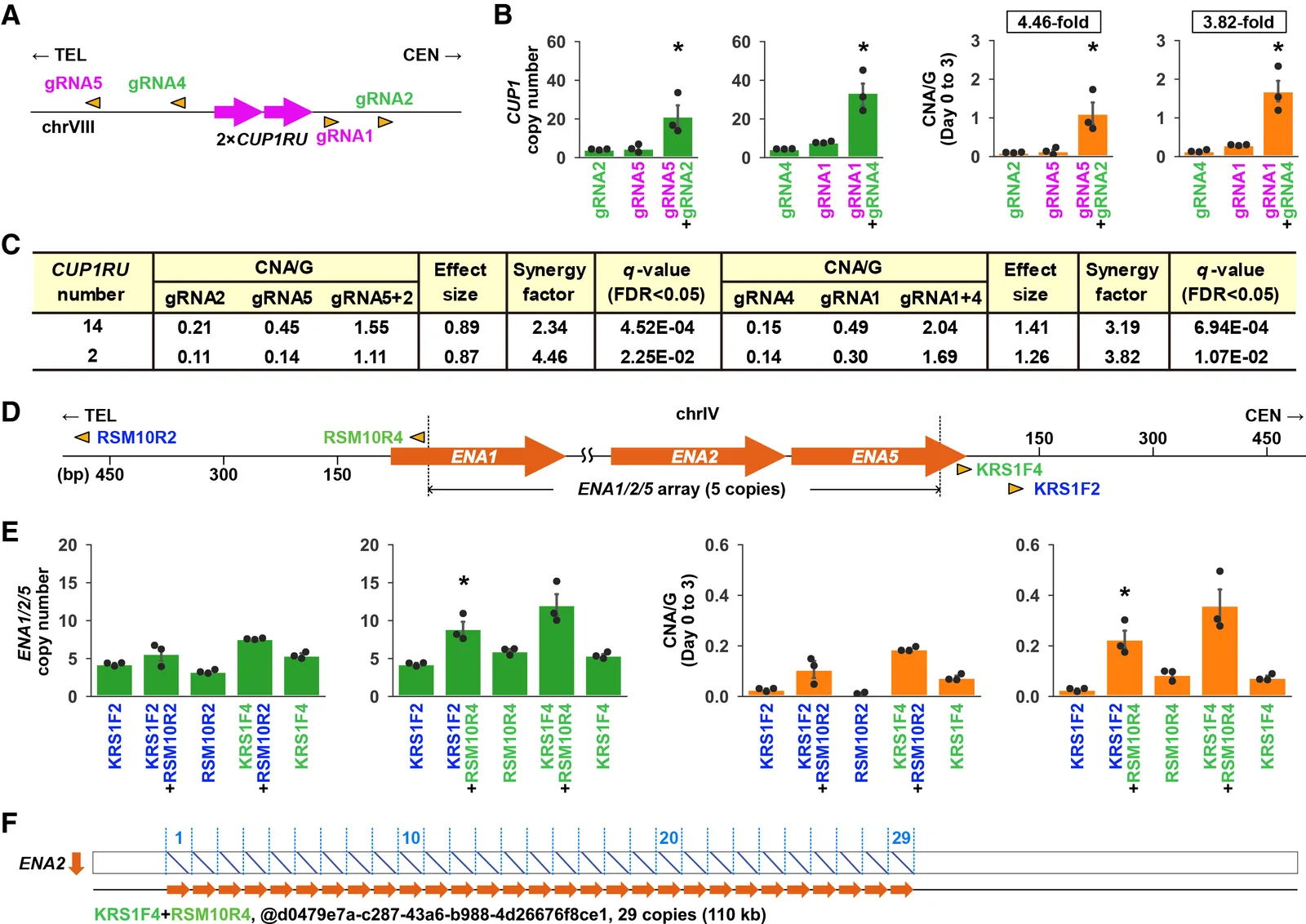
BITREx 2.0-mediated acceleration of expansion-refractory arrays (A) Schematic of the minimal two-unit *CUP1* array. Arrowheads, positions of gRNA-TSs. (B) Expansion of the two-unit *CUP1* array by BITREx 2.0. Two gRNA pairs are used to drive expansion. (Left) *CUP1* copy number at day 3; (right) CNA/G. Error bar, SD (*n* = 3 biological replicates). ∗*q* < 0.05 (Benjamini–Hochberg adjusted). (C) Comparison of BITREx 2.0 efficiency between 14-unit and two-unit *CUP1* arrays. (D) Schematic showing the *ENA1/2/5* locus. Arrowheads, positions of gRNA-TSs. (E) Expansion of the *ENA1/2/5* locus by BITREx 2.0. Four gRNA pairs are used to drive expansion. (Left) *ENA1/2/5* copy number at day 3; (right) CNA/G. Error bar, SD (*n* = 3 biological replicates). ∗*q* < 0.05 (Benjamini–Hochberg adjusted). (F) Structural validation by nanopore sequencing. A representative dot plot compares the *ENA2* reference sequence against a single long read from cells with the KRS1F4+RSM10R4 combination at day 3. For the distribution of estimated *ENA1/2/5* copy numbers, see Figure S5C.

While single nicking with gRNA5 resulted in a 68% reduction in CNA/G on the two-unit array compared to the 14-unit array, this decline was mitigated to just 28% when gRNA5 was paired with gRNA2 in BITREx 2.0 (Figures 6B, 6C, and S5A). Similarly, the 39% reduction observed for single nicking with gRNA1 was attenuated to 17% when combined with gRNA4 (Figures 6B, 6C, and S5A). These buffering effects are attributable to the synergy observed in BITREx 2.0 (4.46- and 3.82-fold for the gRNA5+gRNA2 and gRNA1+gRNA4 combinations, respectively). These results demonstrate the robustness of BITREx 2.0 when targeting low-copy-number arrays, highlighting its ability to efficiently initiate gene amplification even from small starting arrays.

Furthermore, our observation that BITREx 2.0 remains effective for an ARS-less synthetic array (Figure 5E) prompted us to apply the system to the native *ENA1/2/5* locus lacking an internal ARS (Figure 6D). This cluster consists of three to five tandemly arranged copies of three distinct *ENA* paralogs, each encoding a P-type ATPase sodium pump. Although conventional BITREx can drive its expansion, its efficiency remains relatively low; indeed, the *ENA1/2/5* locus represents a challenging target for which no high-efficiency gRNAs have been identified.^11^ This inefficiency is likely exacerbated by several factors: the limited number of repeat units, the absence of an internal ARS, their substantial unit length (∼3.9 kb), and sequence heterogeneity among the repeat units.

To evaluate BITREx 2.0 for the expansion of the native *ENA1/2/5* locus, we tested four gRNA combinations generated by pairing two upstream and two downstream gRNAs (Figures 6E and S5B). In single-nicking BITREx, the CNA/G values for these individual gRNAs ranged from less than 0.01 to 0.09, reflecting the inherent difficulty of expanding this locus. In all tested combinations, BITREx 2.0 showed greater-than-additive CNA/G values. Notably, the pairing of the two most effective gRNAs (yielding 0.09 and 0.07 individually) resulted in a peak performance of 0.36 in the BITREx 2.0 configuration. This enhanced efficacy enabled the longest arrays achieved to date; whereas conventional BITREx for 10 days identified a 19-unit array,^11^ BITREx 2.0 for just 3 days achieved a 29-unit array (Figures 6F and S5C).

Together, these results highlight the utility and versatility of BITREx 2.0 for expanding gene arrays that are otherwise refractory to the original single-nicking BITREx method.

## Discussion

We used strategic targeting of nCas9 to develop two gene amplification methods, namely PNAmp and BITREx. While PNAmp uses a pair of nCas9 to induce a large segmental duplication of up to 1 Mb through a single round of SSA,^8^ the original BITREx exploits iterative ectopic BIR to progressively expand tandem repeats composed of kb-sized units, ultimately generating Mb-sized gene arrays through long-term culture.^11^ In this study, we introduced two strategic improvements to BITREx. First, we developed an ectopic BIR reporter system to expedite the identification of high-performing gRNAs. Next, considering a situation where good gRNAs cannot be identified even with the use of the reporter system, we developed BITREx 2.0 to harness available gRNAs with suboptimal efficiency.

The plasmid-based reporter system depends on the reconstitution of the *ymNeonGreen* gene via ectopic BIR induced by a test gRNA–nCas9 complex (Figure 1). Given the inherent differences in genomic context between a plasmid-based reporter and a native chromosomal locus, we initially questioned whether the plasmid system could accurately predict gRNA efficacy at its chromosomal TSs. However, the reporter system demonstrated robust predictive performance (Figure 2). While the correlation was strong when the distance between the TS and the array was fixed, even the variable-distance configurations provided sufficient predictive power for practical use (Figure S1).

The performance of gRNAs can be predicted by various programs in conventional genome editing but not in BITREx (Figures 2F and S1E). This is presumably because the requirements for good gRNAs in BITREx are more complex than those in conventional genome editing. For instance, efficient amplification requires that (1) the gRNA effectively induces a nick; (2) the gRNA–nCas9 complex ideally remains bound to the target to block nick sealing until replication fork arrival; and (3) the seDSB initiates ectopic rather than orthotopic BIR. Since the efficiency of BITREx is likely determined by these and other unidentified factors, a versatile reporter system is practically useful.

We conceived BITREx 2.0, initially expecting that concurrent nicking at both flanks of a tandem array would produce a simple additive effect relative to single-nicking BITREx (Figure 3A). However, we observed multiple cases where the efficiency of dual nicking far exceeded the sum of the corresponding single-nicking efficiencies in both natural and synthetic arrays, accompanied by a statistically significant interaction between the two gRNAs (Figures 3, 4, 5, and 6). Intriguingly, the effect of BITREx 2.0 does not always scale with the individual efficiencies of the selected gRNAs. In both the natural *CUP1* array (Figure 3B) and the synthetic two-unit *tLEU2* array (Figure S4E), combining two high-efficiency gRNAs resulted in significantly lower performance than combining a high-performing gRNA with a low- or moderate-performing counterpart. Even a gRNA incapable of showing good performance in single-nicking BITREx could contribute to BITREx 2.0 as a potent partner of a high-performance gRNA.

The precise mechanisms underlying this synergy remain to be fully elucidated. While SSA between the first and last repeat units could theoretically drive a near-full duplication of the array (Figure 3A), our genetic analysis did not support this model (Figure 4A). Notably, BITREx 2.0 efficiency was remarkably enhanced in some gRNA pairs in the absence of Rad51 and Rad52, factors essential for canonical BIR and SSA, respectively (Figures 4B and 5B). In this context, it is intriguing to note that the recombinational maintenance of rDNA and *CUP1* arrays has been shown to occur independently of canonical recombination proteins, including Rad51 and Rad52.^22^^,^^23^ Although the molecular basis for this independence remains unclear, our findings suggest that BITREx 2.0 may involve these atypical repair pathways that are characteristic of tandemly repeated genomic loci. Furthermore, BITREx 2.0 proved effective for arrays even lacking an internal ARS, which was originally considered a prerequisite (Figures 5E and 6D). These observations collectively indicate that the mechanism underlying BITREx 2.0 is significantly more complex than initially hypothesized. Rather than adhering to a single, rigid pathway, the system appears to navigate a flexible repair landscape, where the choice of molecular route is dictated by the precise interplay between the two nicks and the host’s genetic environment. Consequently, we propose redefining the acronym BITREx as break-induced tandem repeat expansion, rather than BIR-mediated tandem repeat expansion, to more accurately reflect its broader mechanistic nature.

These two advancements afford significant flexibility in the design of nCas9-mediated gene amplification experiments. When the reporter screen identifies a gRNA with sufficient potency, the original BITREx system can be readily implemented. It should be noted, however, that a high-performing gRNA is not always the most practical choice for applications aiming for a specific, moderate range of copy numbers. For instance, by applying BITREx in a parallelized format to three exogenous carotenogenic genes integrated into different chromosomes, we generated a yeast cell population with diverse gene copy number ratios, from which we identified “elite” genotypes optimized for β-carotene production.^24^ Intriguingly, we found that overamplification of a certain carotenogenic gene exerts an adverse effect on the total yield. Similarly, when examining isogenic clones with different *CUP1* copy numbers generated by BITREx, we observed that those with intermediate copy numbers, rather than the highest, showed the peak copper resistance.^25^ These examples demonstrate that maximal array expansion does not necessarily correlate with optimal phenotypic performance. For such genes, BITREx with intermediate efficiency enables the efficient exploration of meaningful genotypic space.

Conversely, in cases where maximum expansion is the primary objective, or should the initial screen fail to identify candidates with sufficient potency, available gRNAs can be combined to leverage the synergistic power of BITREx 2.0. For applications requiring maximum expansion efficiency, BITREx 2.0 represents the primary choice. Furthermore, the reporter system facilitates the development of a library of high-performing gRNAs from diverse sources. These validated gRNA-TS pairs could be strategically co-integrated with synthetic arrays or utilized to re-engineer the flanking regions of natural arrays—where sequence modification is permissible—to compensate for a lack of optimal endogenous TSs (Figures 3D and S2B).

In conclusion, we expect these enhancements to significantly improve the utility and reliability of BITREx across a broad spectrum of genomic applications, from basic research to advanced biotechnology.

### Limitations of the study

First, the reporter system is effective only below fluorescence saturation and should therefore be regarded primarily as a screening and ranking tool rather than a quantitative measure under all conditions. It has been validated only for single nicking and not for dual nicking. Second, although we demonstrated the effectiveness of BITREx 2.0, the molecular basis of its synergistic effects is not yet understood. Furthermore, the influences of array length, sequence context, and chromatin environment on BITREx 2.0 efficiency, as well as the potential applicability of splint DNA^11^ to this platform, warrant further investigation. Finally, the performance of the reporter system and BITREx 2.0 in organisms other than *S*. *cerevisiae* has not yet been evaluated.

## Resource availability

### Lead contact

Further information and requests for resources and reagents should be addressed to and will be fulfilled by the lead contact, Takashi Ito (ito.takashi.352@m.kyushu-u.ac.jp).

### Materials availability

Requests for the generated plasmids and strains in this study should be directed to the lead contact, Takashi Ito (ito.takashi.352@m.kyushu-u.ac.jp).

### Data and code availability

• All raw sequencing data used in this study are deposited in the DDBJ BioProject database: PRJDB40436.
• All original codes used in this study are available from Zenodo at https://doi.org/10.5281/zenodo.18440611.
• Any additional information required to reanalyze the data reported in this paper is available from the lead contact upon request.

## Acknowledgments

We thank the technical support from the Research Support Center of the Research Center for Human Disease Modeling at 10.13039/501100004096Kyushu University Graduate School of Medical Sciences, which is partially supported by the Mitsuaki Shiraishi Fund for Basic Medical Research. This work was supported by JST CREST grant no. JPMJCR19S1 (T.I.) and 10.13039/501100001691JSPS KAKENHI grant nos. JP24K02015 (T.I.) and JP25K00104 (H.T.).

## Author contributions

Conceptualization, data curation, resources, and writing – review and editing, H.T., S.O., and T.I.; formal analysis and writing – original draft, H.T.; funding acquisition, H.T. and T.I.; investigation, methodology, software, validation, and visualization, H.T. and S.O.; project administration and supervision, T.I.

## Declaration of interests

The authors declare no competing interests.

## Declaration of generative AI and AI-assisted technologies in the writing process

During the preparation of this work, the authors used ChatGPT and Gemini to improve readability of sentences. After using this tool or service, the authors reviewed and edited the content as needed and take full responsibility for the content of the publication.

## STAR★Methods

### Key resources table

**Table:** 

REAGENT or RESOURCE	SOURCE	IDENTIFIER
**Chemicals, peptides, and recombinant proteins**		

17β-Estradiol	Nacalai tesque	Cat# 14541-74
Doxycycline hydrochloride	Apollo scientific	Cat# BID0121
Hygromycin B	Nacalai tesque	Cat# 07296-66

**Critical commercial assays**		

KOD One® PCR Master Mix (Dye-free 2×PCR Master Mix)	TOYOBO	Cat# KMM-101
KOD SYBR® qPCR Mix	TOYOBO	Cat# QKD-201
Chelex 100 Chelating Resin, biotechnology grade, 100–200 mesh, sodium form	Bio-Rad	Cat# 1432832
Quick-DNA Fungal/Bacterial Miniprep Kit	ZYMO RESEARCH	Cat# D6005
Monarch HMW DNA Extraction Kit for Tissue	NEB	Cat# T3060L
NEB Golden Gate Assembly Kit (BsaI-HF v2)	NEB	Cat# E1601L
NEBuilder HiFi DNA Assembly Master Mix	NEB	Cat# E2621L
Ligation Sequencing Kit	Oxford Nanopore Technologies	SQK-LSK114
Native Barcoding Kit 96 V14	Oxford Nanopore Technologies	SQK-NBD114.96
PromethION Flow Cell (R10.4.1)	Oxford Nanopore Technologies	FLO-PRO114M

**Deposited data**		

*S. cerevisiae* S288C reference genome: sacCer3	Saccharomyces Genome Database	https://www.ncbi.nlm.nih.gov/datasets/genome/GCF_000146045.2/
Escherichia phage lambda, complete genome	GenBank	Accession no. J02459https://www.genome.jp/dbget-bin/www_bget?genbank-phg:J02459
Raw sequence data	This paper	DDBJ BioProject database: PRJDB40436

**Experimental models: Organisms/strains**		

*S. cerevisiae*: Strain background: BY4741	Brachmann et al.^26^	N/A
All other synthetic yeast strains used in this paper, listed in Table S1	This paper	N/A

**Oligonucleotides**		

All oligonucleotides used in this paper, listed in Table S4	This paper	N/A

**Recombinant DNA**		

All plasmids used in this paper, listed in Table S2	This paper	N/A

**Software and algorithms**		

YeastMate	Bunk et al.^27^	https://github.com/hoerlteam/YeastMate
ImageJ/Fiji	Schindelin et al.^28^	https://imagejnet.software/fiji/
MinKNOW	Oxford Nanopore Technologies	https://community.nanoporetech.com/downloads?from=support
Dorado v0.7.3	Oxford Nanopore Technologies	https://community.nanoporetech.com/downloads?from=support
NanoPlot	De Coster et al.^29^	https://github.com/wdecoster/NanoPlot
samtools v1.10	Danecek et al.^30^	https://github.com/samtools/samtools
bedtools v2.27.1	Quinlan and Hall^31^	https://github.com/arq5x/bedtools2
Bedgraph_norm_ratio.py	Satoshi Okada	https://doi.org/10.5281/zenodo.11515696
IGVtools v 2.16.2	Robinson et al.^32^	https://igv.org/
Minimap2 v2.17-r941	Li^33^	https://github.com/lh3/minimap2
BLAST	Altschul et al.^34^	https://blast.ncbi.nlm.nih.gov/blast/Blast.cgi
YASS	Noé and Kucherov^35^	https://bioinfo.univ-lille.fr/yass/index.php
Nanopore_for_gene_array-v1.0.0.	Hiroaki Takesue	https://doi.org/10.5281/zenodo.18440610

### Experimental model and study participant details

The budding yeast *Saccharomyces cerevisiae* was used as the primary experimental model in the study. The haploid yeast strain BY4741 (*MAT***a** *his3*Δ*1 leu2*Δ*0 met15*Δ*0 ura3*Δ*0*) was used as the parental strain.^26^

### Method details

#### Yeast strain construction

All yeast strains used in this study are listed in Table S1. They were constructed using standard yeast genetic techniques, including transformation with linearized integrative plasmids (Table S2) or PCR products (Table S3), as well as genome editing using appropriate gRNAs (Table S4) with a previously described vector series.^36^

The gene encoding the artificial transcription factor GEV^13^ under the control of the *CUP2* promoter (*pCUP2*) was integrated at the *pCUP2* on chromosome VII via plasmid integration with *HphMX* marker, as described previously.^11^ The gene encoding nCas9 (*Streptococcus pyogenes* Cas9^D10A^), under the control of the *GAL1* promoter (*pGAL1*), was integrated at the *pGAL1* on chromosome II via plasmid integration with *LEU2* marker, as described previously.^11^

#### Plasmid construction

To express gRNAs (Table S4) under the control of the *GAL1* promoter (*pGAL1*), centromeric plasmids carrying the *URA3* marker (Table S2) were utilized. Each gRNA sequence was flanked by hammerhead and HDV ribozymes at the 5′ and 3′ ends, respectively, enabling precise self-excision from the primary transcript generated by RNA polymerase II. For the simultaneous expression of two gRNAs in BITREx 2.0, we constructed plasmids containing two copies of the expression cassettes in a head-to-head orientation (Table S2).

To construct the reporter plasmid, the gRNA expression vector was further modified to harbor a split *ymNeonGreen* reporter system. This unit consists of an *nGreen* segment, a *TDH3* promoter (*pTDH3*), an *ymNeonG* segment, and a pair of BsaI sites for the seamless cloning of the gRNA target sequences.

#### Induction of BITREx/BITREx 2.0

Yeast cells were grown at 30°C overnight in 300 μL of SC−Ura or SC−Ura−Leu medium supplemented with 2% glucose. Where required, the medium was further supplemented with 500 ng/mL Dox and/or 200 μg/mL hygromycin B. On the following day, the OD_620_ of each sample was recorded, and 0.5–30 μL of the culture diluted up to 1 × 10^6^-fold was inoculated into 300 μL of the fresh medium containing 10 nM β-estradiol. The division number per day was calculated from the change of OD_620_.

#### Fluorescence microscopy and image processing

Image acquisition of yeast cells was performed on an imaging system (EVOS M7000, Thermo Fisher Scientific, Waltham, MA) with a 20× objective lens (UCPlanFL *N* 20×, 51897b, Olympus, Tokyo, Japan). Images were acquired using the integrated CMOS camera and illumination system controlled via the EVOS software. Z-stacks were 21 × 1.0 μm. A YFP filter set (EVOS Light Cube, YFP 2.0, AMEP4954, Thermo Fisher Scientific) was used to acquire mNeonGreen fluorescence images, with the excitation light power set to 1 × 10^−5^ (arbitrary units; EVOS software). Bright-field images were captured only at the middle position of the Z-stacks.

Bright-field images were analyzed using YeastMate^27^ to segment individual yeast cells and generate region of interest (ROI) masks. Image processing and analysis were performed in Fiji/ImageJ.^28^ To generate two-dimensional fluorescence images from Z-stacks, background subtraction (sliding paraboloid radius set at 5 pixels with disabled smoothing) was applied, followed by maximum-intensity projection of all 21 Z-slices. Maximum-projected fluorescence images and corresponding YeastMate-derived ROI images were superimposed.

To define mNeonGreen-positive cells, binary cell-area masks were first generated from the YeastMate-derived ROI images. The masks were applied to the maximum-projected mNeonGreen fluorescence images. A fixed threshold was then set at pixel intensities greater than 100 (arbitrary units in Fiji/ImageJ). With this threshold active, “Analyze Particles” was run to detect fluorescent objects, restricting the area to 25–1500 pixels. Particles detected under these conditions were counted as mNeonGreen-positive cells. ΔmNeonGreen(+) was calculated using the following formula: ΔmNeonGreen(+) = (mNeonGreen-positive cell ratio)_β-estradiol(+)_ – (mNeonGreen-positive cell ratio)_β-estradiol(−)_. Integrated density values were calculated for each cell from the masked mNeonGreen fluorescence images. Representative images were prepared for figures by global adjustment of brightness and contrast and cropping; these adjustments were not used for quantitative analyses.

#### Quantitative PCR

Genomic DNA extracted using the GC prep method^37^ was diluted 10-fold with distilled water before qPCR. Each qPCR solution (20 μL) contained 2 μL of diluted DNA, 10 μL of KOD SYBR qPCR Mix (TOYOBO), 0.04 μL of 50× ROX Reference Dye (TOYOBO), 2 pmol each of the forward and reverse primers (Table S3). Each qPCR assay was performed in duplicate, using QuantStudio3 (Applied Biosystems) according to the manufacturer’s instructions. The amplification condition was initial denaturation at 98°C for 2 min followed by 40 times iteration of a 3-step thermal cycle composed of 98°C for 10 s, 55°C for 10 s, and 68°C for 30 s. All qPCR runs included 10-fold serial dilutions to generate standard curves. The quantity of target genes was normalized to that of *ACT1*. The copy number of target genes in the standard curves was calibrated by nanopore sequencing results in the BY4741 strain.

The copy number alteration per generation (CNA/G) for each target gene was calculated using the following formula:

$$CNA/G=\frac{{{CN}_{day3}-CN}_{day0}}{n}$$

where *CN*_*day*3_ and *CN*_*day*0_ represent the absolute copy numbers estimated by qPCR at day 3 and day 0, respectively, and *n* denotes the number of cell divisions during the period, estimated from OD_620_ values.^11^

The synergy factor for BITREx 2.0 using gRNA#1 and gRNA#2 was calculated with the following formula:

$$Synergy\;factor=\frac{{CNA/G}_{gRNA\#1+gRNA\#2}}{{CNA/G}_{gRNA\#1}+{CNA/G}_{gRNA\#2}}$$

If the two ectopic BIR events occur independently, the synergy factor would be equal to 1.0. If ectopic BIR at one end of the array adversely affects that at the other end, the synergy factor would be expected to be less than 1.0. Conversely, a synergy factor greater than 1.0 suggests a positive synergistic interaction between the two ectopic BIR events. We similarly calculated synergy factors using the absolute copy number and CNA.

For evaluation of interactions between two gRNAs in BITREx 2.0, two-way ANOVA was performed using data with TEF1 gRNA as a control. *p*-values were adjusted for multiple testing using the Benjamini–Hochberg procedure to control the false discovery rate (FDR), and adjusted *p*-values (*q*-values) were reported. Findings were considered statistically significant when *q* < 0.05.

#### Nanopore sequencing and data analysis

Genomic DNA was extracted using Monarch HMW DNA Extraction Kit for Tissue (NEB). We avoided vortexing to obtain high molecular weight DNA and used mixing by gentle pipetting with a wide-bore tip. Plasmid DNA extraction from yeast cells was performed as described previously.^38^ DNA libraries for nanopore whole-genome sequencing were prepared using the ligation sequencing kit SQK-LSK114 and the native barcoding kit SQK-NBD114 (Oxford Nanopore Technologies) according to the manufacturer’s instructions. We modified the protocol of the ligation sequencing kit as follows: DNA fragmentation, omitted; duration of the enzymatic repair steps at 20°C and 65°C, both extended from 5 min to 30 min; and the duration of the ligation step, extended from 10 min to 30 min; incubation time for elution with 0.4× AMPure XP, extended from 10 min to 20 min. The library was sequenced with the flowcell FLO-PRO114M R10.4.1 using a PromethION 2 Solo sequencer (Oxford Nanopore Technologies). MinKNOW software was used to control the PromethION device. The run time was set to 72 h. Base calling was performed using Dorado v0.7.3. The assessment of sequencing data was performed using NanoPlot.^29^

We used nanopore sequencing data in FASTQ format and mapped reads to the S288c reference genome (version R64-2-1, http://sgd-archive.yeastgenome.org/sequence/S288C_reference/genome_releases/S288C_reference_genome_R64-2-1_20150113.tgz) using SAMtools^30^ and BEDtools,^31^ and then normalized read count of each nucleotide was calculated using Bedgraph_norm_ratio.py (https://doi.org/10.5281/zenodo.11515696). Data were visualized with the IGV.^32^ To eliminate the effect of read clipping and achieve a more accurate estimation of repeat unit number, we collected all reads containing the repeat unit using Minimap2.^33^ We then used the reference sequence of a target gene as a query in a BLAST^34^ search against the collected reads and estimated the copy number based on the number of BLAST hits, as described previously.^11^

We used nanopore sequencing data in FASTA format to draw dot plots using YASS.^35^ We first selected reads spanning the entire array using 1-kb upstream and downstream sequences of the target array as queries of Minimap2^33^ and then used these reads as the first input sequence for YASS. As the second input, we used the reference sequence of the repeat unit. By manually counting the diagonal lines in each dot plot, we determined the copy number of the repeat unit.

We have made our pipeline for targeted nanopore sequencing of tandem gene arrays publicly available (https://doi.org/10.5281/zenodo.18440610).

### Quantification and statistical analysis

Quantification of gene copy and division numbers was performed as described above using three or four independent biological replicates (*n* = 3 or 4) per strain.

Statistical analysis was performed using SciPy (1.16.3) in Python (3.13.9). For comparisons of copy numbers and CNA/G values between two groups, unpaired two-tailed Student’s *t* test was used to determine statistical significance, assuming normal distribution, with *p* < 0.05 considered statistically significant (Figures 4C and 5B; Figures S3D, and S4E). For evaluation of fluorescence intensity, Mann–Whitney U test was used (Figure 5D). For evaluation of interactions between two gRNAs in BITREx 2.0, two-way ANOVA was performed. *p*-values were adjusted using the Benjamini–Hochberg procedure to control the FDR, and results with *q* < 0.05 were considered statistically significant (Figures 3B–3D, 5F, 6B, 6C, 6E; Figures S2A, S2B, S4G, S5A, and S5B).

Data visualization was conducted using the matplotlib (3.10.6) and seaborn (0.13.2) libraries in Python (3.13.9). Bar graphs for copy numbers at day 3 and the mean CNA/G values display the mean ± SD, with individual data points overlaid to ensure data transparency.

## Supplemental information

Document S1. Figures S1–S5

Table S1. Yeast strains used in this study, related to Figures 1–6 and S1–S5

Table S2. Plasmids used in this study, related to Figures 1–6 and S1–S5

Table S3. Oligonucleotides used in this study, related to Figures 1–6 and S1–S5

Table S4. gRNAs used in this study, related to Figures 1–6 and S1–S5

Document S2. Article plus supplemental information
