# Supplementary material for: Strategic enhancement of Cas9 nickase-driven gene amplification
Source: Cell Rep Methods. 2026 Aug 6;6(10):101544. doi: 10.1016/j.crmeth.2026.101544 (PMC13728499; doi:10.1016/j.crmeth.2026.101544)
Supplement: Supplementary file 1 [file mmc1.pdf]

Cell Reports Methods, Volume *6*

Supplemental information

Strategic enhancement of Cas9

nickase-driven gene amplification

Hiroaki Takesue, Satoshi Okada, and Takashi Ito

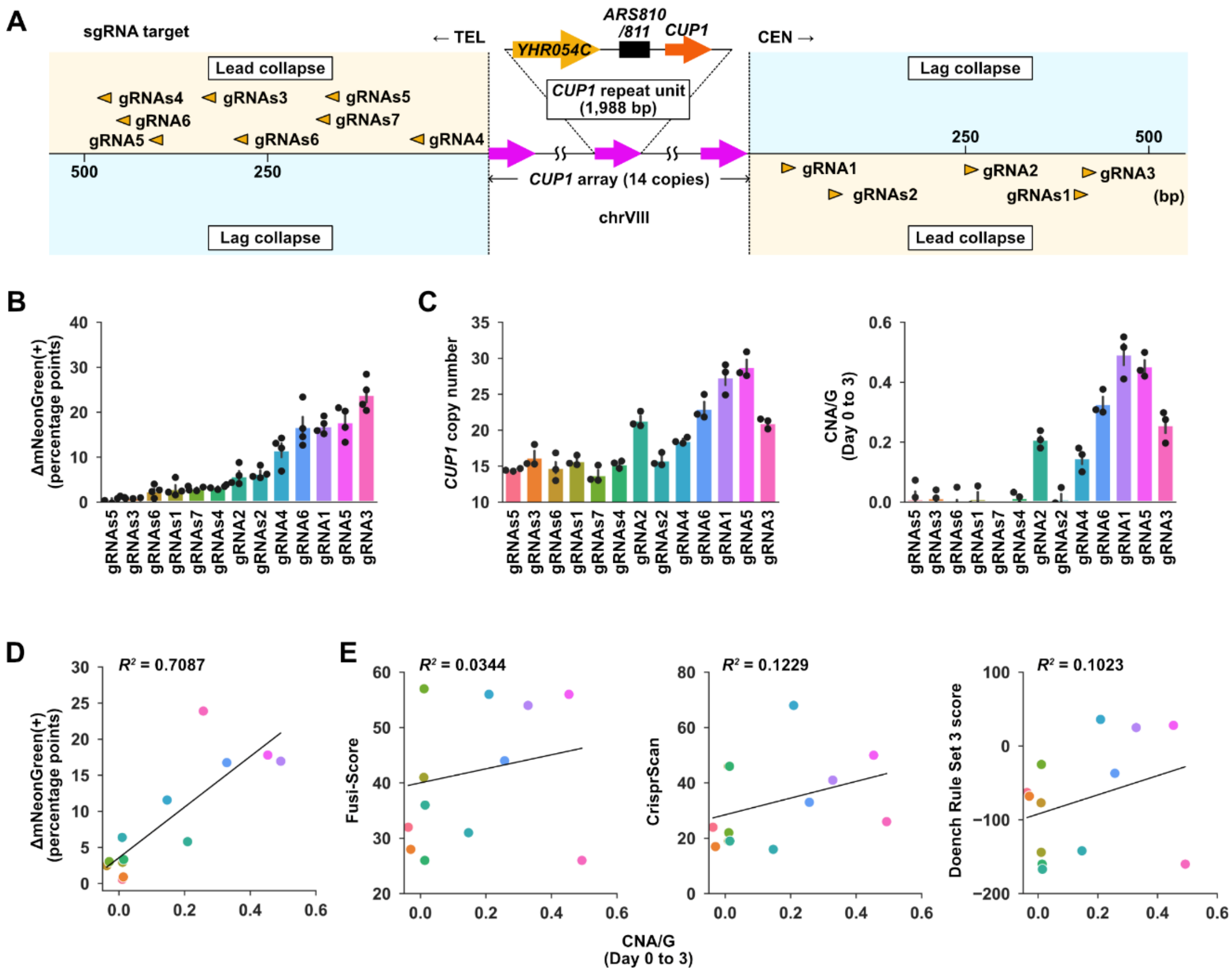

Figure S1: Validation of the ectopic BIR reporter system for predicting gRNA performance, related to Figure 2

(A) Schematic showing the *CUP1* locus, with arrowheads indicating the positions of 13 gRNA-TSs.

(B) Evaluation of reporter activities on episomal target sites. Efficiency is quantified as ΔmNeonGreen(+) (percentage points), the increased frequency of mNeonGreen-positive cells after 24 hours of BITREx induction with β-estradiol, compared with the β-estradiol(−) condition. Error bar, SD (n = 4 biological replicates).

(C) Performance of λ-derived gRNAs at chromosomal loci. (Left) *CUP1* copy number at day 3; (Right) CNA/G. Error bar, SD (n = 3 biological replicates).

(D) Correlation between episomal and chromosomal performance. Linear regression shows the relationship between reporter activities (ΔmNeonGreen(+)) and CNA/G values of *CUP1* array expansion.

(E) Predictive power of computational gRNA design tools. For each gRNA, Doench/Fusi score (Rule Set 2 on-target score),[14] CRISPRscan score,[15] and Doench Rule Set 3 score[16] are plotted against its CNA/G value.

A

gRNAs		$CN_{day\ 3}$			Effect size	Synergy factor	*q*-value (FDR<0.05)
#1	#2	#1	#2	#1+#2			
s5	s2	14.47	15.83	22.71	9.21	0.75	5.80E-04
4	2	18.55	21.38	26.01	2.89	0.65	9.13E-02
5	1	28.83	27.36	34.69	-4.69	0.62	9.45E-02
s2	5	15.83	28.83	49.32	21.47	1.10	4.40E-03
s5	1	14.47	27.36	36.54	11.51	0.87	2.36E-03
2	5	21.38	28.83	55.42	22.01	1.10	7.61E-04
4	1	18.55	27.36	73.25	44.15	1.60	1.03E-04

B

Genotype		CNA/G			Effect size	Synergy factor	*q*-value (FDR<0.05)	CNA/G			Effect size	Synergy factor	*q*-value (FDR<0.05)
HIS3 insertion	gRNA-TS	gRNA4	λ or 1	pair				gRNA5	λ or 1	pair			
−	1	0.15	0.49	2.04	1.41	3.19	1.19E-03	0.45	0.49	0.68	-0.26	0.72	2.10E-02
+	1	0.32	0.96	2.11	0.84	1.65	4.85E-02	0.57	0.96	1.86	0.34	1.22	4.33E-02
	λR8	0.92	0.55	0.40	-1.06	0.27	1.75E-02	0.74	0.55	3.58	2.30	2.79	3.35E-03
	λL8	0.24	0.15	0.64	0.26	1.64	5.63E-02	0.90	0.15	2.26	1.22	2.15	4.68E-02
	λR4	0.42	0.40	1.68	0.87	2.05	1.81E-02	0.58	0.40	4.26	3.29	4.34	6.70E-04
	λL3	0.67	0.25	1.74	0.83	1.90	1.84E-03	1.07	0.25	2.87	1.56	2.18	2.26E-02
	λR7	0.46	0.49	-0.01	-0.95	-0.01	1.27E-02	0.61	0.49	5.99	4.90	5.47	3.47E-03
	λR3	0.33	0.59	1.84	0.92	1.99	2.17E-02	0.67	0.59	6.14	4.88	4.86	3.82E-04
	λR5	0.45	0.71	1.58	0.42	1.36	2.34E-01	0.60	0.71	5.15	3.84	3.92	2.54E-03
	λL7	0.55	0.70	1.17	-0.07	0.94	2.60E-01	1.13	0.70	8.79	6.97	4.81	1.69E-02
	λL2	0.45	0.89	1.04	-0.30	0.77	1.82E-01	0.90	0.89	10.32	8.53	5.75	2.22E-02
	λL1	0.41	0.85	0.46	-0.80	0.36	9.95E-03	0.58	0.85	-0.05	-1.47	-0.03	2.49E-03
	λL6	0.43	0.87	0.46	-0.84	0.35	2.24E-02	1.15	0.87	2.19	0.18	1.09	3.76E-01
	λL5	0.42	0.96	1.39	0.02	1.01	4.14E-01	0.91	0.96	3.66	1.80	1.96	1.03E-01
	λR2	0.40	1.12	2.94	1.44	1.94	1.14E-02	0.90	1.12	13.29	11.28	6.59	6.91E-04

Genotype		$CN_{day\ 3}$			Effect size	Synergy factor	*q*-value (FDR<0.05)	$CN_{day\ 3}$			Effect size	Synergy factor	*q*-value (FDR<0.05)
HIS3 insertion	gRNA-TS	gRNA4	λ or 1	pair				gRNA5	λ or 1	pair			
−	1	18.55	27.36	73.25	44.15	1.60	1.03E-04	28.83	27.36	34.69	-4.69	0.62	6.01E-02
+	1	21.60	35.01	43.21	2.69	0.76	2.56E-01	28.65	35.01	55.07	7.50	0.87	9.46E-02
	λR8	43.88	32.39	28.15	-32.03	0.37	4.17E-03	38.92	32.39	96.20	40.98	1.35	1.22E-02
	λL8	18.19	15.59	25.57	7.88	0.76	2.09E-02	35.67	15.59	53.38	18.21	1.04	7.83E-02
	λR4	32.67	32.22	58.76	9.96	0.91	8.99E-02	36.98	32.22	98.00	44.89	1.42	1.85E-03
	λL3	34.91	23.54	53.77	11.41	0.92	5.52E-03	45.80	23.54	79.17	25.92	1.14	6.28E-02
	λR7	33.50	35.97	21.25	-32.13	0.31	7.43E-03	37.51	35.97	141.22	83.84	1.92	1.19E-02
	λR3	27.17	34.20	58.18	12.90	0.95	7.07E-02	36.35	34.20	124.40	69.94	1.76	1.40E-03
	λR5	30.84	37.93	52.70	0.02	0.77	4.34E-01	34.86	37.93	109.22	52.51	1.50	9.95E-03
	λL7	29.53	33.31	40.70	-6.05	0.65	6.76E-02	44.93	33.31	162.57	100.42	2.08	3.03E-02
	λL2	27.77	39.23	35.85	-15.05	0.54	2.52E-02	39.41	39.23	163.10	100.55	2.07	5.82E-02
	λL1	27.55	38.94	26.27	-24.13	0.40	5.39E-03	31.95	38.94	15.90	-38.91	0.22	4.67E-03
	λL6	28.40	39.77	26.20	-25.88	0.38	8.61E-03	46.86	39.77	57.05	-13.50	0.66	2.06E-01
	λL5	26.53	40.91	43.83	-7.53	0.65	7.31E-02	39.72	40.91	72.12	7.57	0.89	3.19E-01
	λR2	27.73	46.44	70.32	12.24	0.95	6.65E-02	40.84	46.44	194.55	123.36	2.23	3.10E-03

C

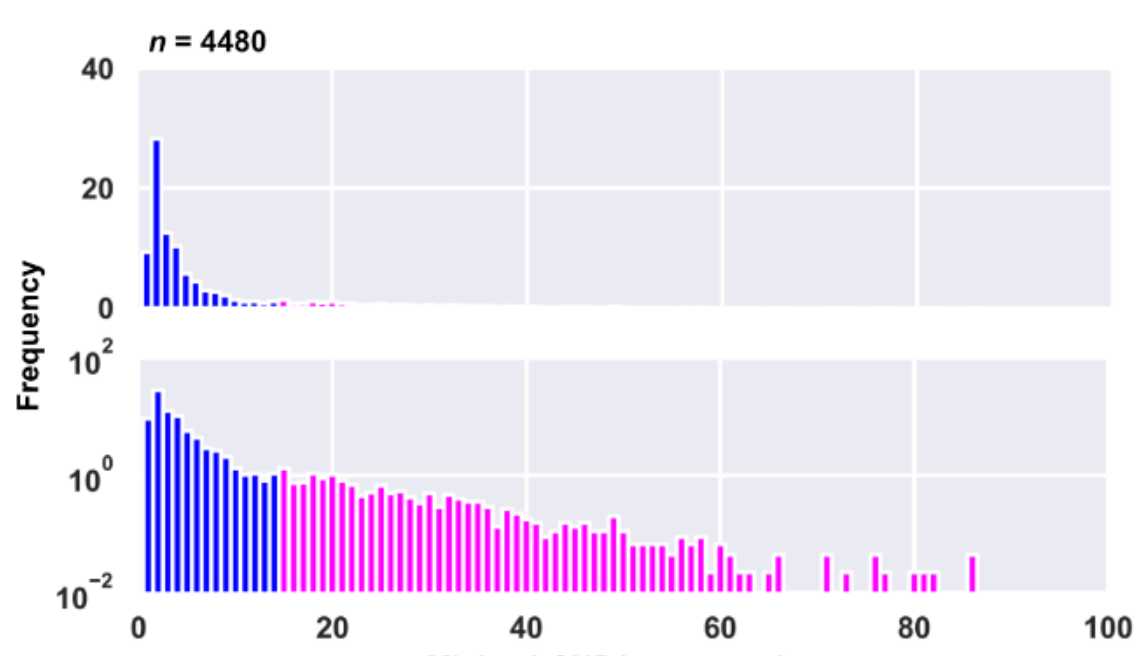

D

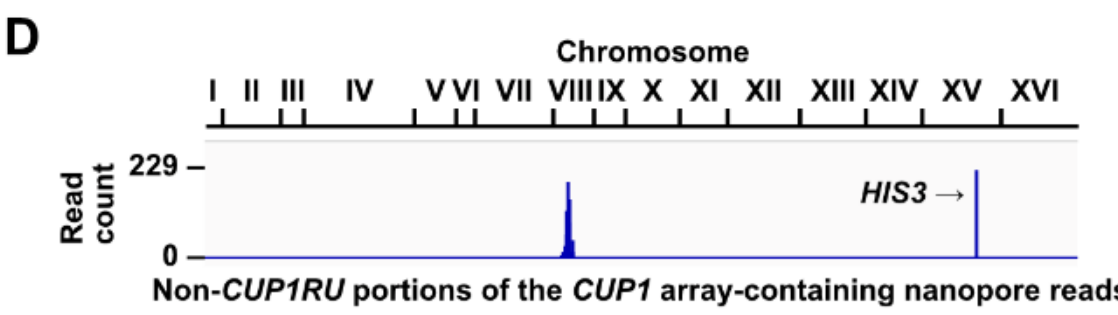

E

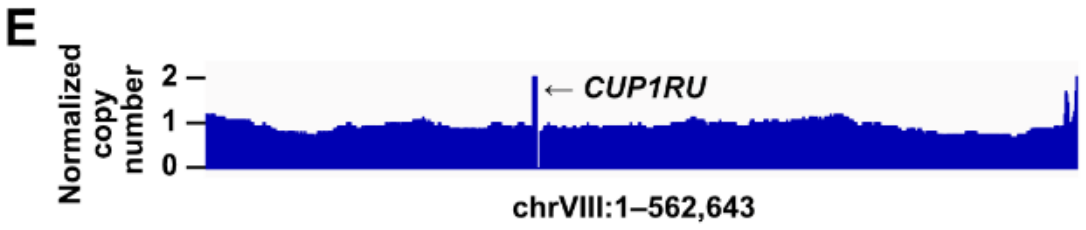

(*legend on next page*)

Figure S2. BITREx 2.0 applied to *CUP1* arrays, related to Figure 3

(A) Effects of BITREx 2.0 on absolute copy number of the endogenous *CUP1* array. Data are presented similarly to Figure 3C but summarized for copy number at day 3 ($CN_{day\ 3}$) instead of CNA/G.

(B) Effects of BITREx 2.0 on modified *CUP1* arrays. We combine gRNA4 or gRNA5 (targeting the telomere-proximal flank) with one of the 13 λ-derived gRNAs or the original gRNA1 (targeting the centromere-proximal flank) across 14 engineered strains. Each strain harbors a specific λ-derived gRNA-TS or the original gRNA1-TS, paired with a *HIS3* marker integrated 126 bp downstream (Figure 2B). Data are presented similarly to Figure 3C but summarized for both CNA/G and CN. Note that the control strain retaining the original gRNA1-TS but carrying the *HIS3* marker insertion shows significantly altered efficiencies for gRNA1, gRNA4, and gRNA5. This *HIS3* insertion may delay the arrival of the inward-migrating replication fork, initiated from *ARS809* (the nearest neighboring origin in the centromere-proximal flank), at the gRNA1-TS. This delay is likely attributable to both the increased physical distance between *ARS809* and the TS and the potential deceleration of the replication fork by counteracting *HIS3* transcription. Such a delay would increase the likelihood of the nick being traversed by the outward-migrating fork originating from *ARS810/811* within the *CUP1* array, thereby facilitating BITREx at this site. However, it remains unclear how this insertion-mediated alteration in the genomic environment influences the performance of gRNA4 and gRNA5, which target the opposite flank of the array. Furthermore, even with the shared *HIS3* insertion, the performance of gRNA4 and gRNA5 exhibits substantial variation across the 13 strains bearing different λ-derived TSs. The mechanisms underlying these variations also remain elusive, highlighting the complex, context-dependent nature of BITREx and suggesting that its efficiency is governed by intricate genomic factors that preclude a simple, unified explanation.

(C) Distribution of *CUP1* copy number estimated from nanopore sequencing data. Data are obtained from cells with gRNA5 and gRNAλR2. The top and bottom panels display the vertical axis on a linear scale and a logarithmic scale, respectively, to facilitate the visualization of highly expanded arrays. Note that spanning entire expanded arrays becomes increasingly challenging as their length grows. To address this technical constraint, we have adopted a conservative approach by counting the *CUP1RU* sequence within each read to provide a minimal estimate of the array length, rather than relying solely on a few exceptionally long reads. Among the 4,480 reads containing at least one *CUP1RU*, 746 reads (16.7%; indicated in magenta) are identified to carry 15 or more copies (i.e., classified as expanded). Considering the inherent read truncation within the *CUP1* array, this value represents the most conservative estimate; thus, the actual proportion of cells undergoing BITREx-mediated expansion likely exceeds 16.7%.

(D) Chromosomal distribution of *CUP1* array-flanking sequences in the strain subjected to BITREx 2.0 using gRNA5 and gRNAλR2. From 26,265 nanopore reads containing *CUP1RU*, their non-*CUP1RU* portions are mapped to the reference genome sequence. The peak on chromosome VIII corresponds to the endogenous *CUP1* locus, while the peak on chromosome XV reflects the *his3Δ1* locus, which retains residual homology with the *HIS3* marker integrated in the centromere-proximal flank of the *CUP1* array.

(E) Normalized read counts across chromosome VIII in nanopore sequencing of the strain subjected to BITREx 2.0 using gRNA5 and gRNAλR2. Read counts are normalized to the average counts of genomic regions excluding rDNA, *CUP1RU*, Ty elements, and mitochondrial DNA. Consequently, the normalized read count directly indicates the copy number. Note that while the sacCer3 reference genome sequence contains two copies of *CUP1RU*, the second copy is masked with “N” prior to mapping. The gap in the read counts adjacent to *CUP1RU* is due to the masking. The average read depth of chromosome VIII was comparable to the genome-wide average of 189, except for the *CUP1* locus and the polymorphic subtelomeric regions.

A

Genotype	gRNA2	gRNA5	gRNA5+s2	gRNA5+2	gRNA4	gRNA1	gRNA1+s5	gRNA1+4
wild-type	26.55	27.73	26.00	25.34	26.95	27.06	25.17	26.70
*pol32*Δ	13.40	15.98	14.96	11.00	17.07	16.92	16.99	12.77
pif1-m2	19.38	22.04	21.91	17.25	23.65	23.66	23.64	19.75
*rad51*Δ	14.87	12.32	12.62	12.01	17.58	17.89	17.53	11.15
*rtt109*Δ	11.96	11.47	12.28	10.15	14.11	14.21	14.13	11.18
*rad52*Δ	13.91	14.33	11.13	8.58	18.49	19.02	17.35	9.72
*rad1*Δ	18.56	23.19	23.00	17.78	23.44	23.35	23.39	19.16
*msh2*Δ	18.15	22.74	21.44	17.63	23.12	23.41	23.23	19.43
*msh3*Δ	18.24	22.92	21.76	18.14	23.28	23.10	23.15	19.43

B

	gRNA2			gRNA5			gRNA5+s2			gRNA5+2		
	CNA	1/G	CNA/G	CNA	1/G	CNA/G	CNA	1/G	CNA/G	CNA	1/G	CNA/G
wild-type	1.00	1.00	1.00	1.00	1.00	1.00	1.00	1.00	1.00	1.00	1.00	1.00
*pol32*Δ	-1.43	1.98	-2.95	-0.81	1.73	-1.43	-0.32	1.74	-0.55	-0.25	2.30	-0.59
pif1-m2	0.65	1.37	0.89	-0.30	1.26	-0.37	0.03	1.19	0.04	0.60	1.47	0.87
*rad51*Δ	-0.72	1.79	-1.28	0.14	2.25	0.32	0.07	2.06	0.15	5.25	2.19	11.57
*rtt109*Δ	6.68	2.25	15.06	2.54	2.42	6.15	1.26	2.12	2.65	3.18	2.46	7.81
*rad52*Δ	1.07	1.91	2.11	0.20	2.25	0.45	0.08	2.34	0.21	2.32	2.88	6.66
*rad1*Δ	0.38	1.43	0.55	0.02	1.20	0.02	0.16	1.13	0.18	0.54	1.43	0.76
*msh2*Δ	0.10	1.46	0.15	-0.37	1.22	-0.46	0.11	1.21	0.13	0.46	1.44	0.65
*msh3*Δ	-0.75	1.46	-1.10	-0.19	1.21	-0.23	0.24	1.19	0.29	0.50	1.40	0.71

	gRNA4			gRNA1			gRNA1+s5			gRNA1+4		
	CNA	1/G	CNA/G	CNA	1/G	CNA/G	CNA	1/G	CNA/G	CNA	1/G	CNA/G
wild-type	1.00	1.00	1.00	1.00	1.00	1.00	1.00	1.00	1.00	1.00	1.00	1.00
*pol32*Δ	-1.19	1.58	-1.92	-0.47	1.64	-0.77	-0.47	1.48	-0.70	-0.16	2.09	-0.35
pif1-m2	-0.30	1.14	-0.34	0.17	1.17	0.19	0.16	1.06	0.17	0.45	1.35	0.62
*rad51*Δ	-0.20	1.53	-0.30	0.21	1.55	0.32	0.09	1.44	0.13	0.31	2.27	0.72
*rtt109*Δ	4.56	1.91	8.77	5.02	1.95	9.80	4.05	1.78	7.29	2.03	2.37	4.89
*rad52*Δ	1.12	1.46	1.65	1.32	1.46	1.92	0.50	1.45	0.74	0.29	2.68	0.77
*rad1*Δ	0.46	1.15	0.54	0.68	1.19	0.81	0.87	1.08	0.94	0.61	1.39	0.87
*msh2*Δ	1.08	1.17	1.27	0.71	1.19	0.84	0.74	1.08	0.82	0.72	1.37	1.00
*msh3*Δ	-0.30	1.16	-0.34	0.54	1.20	0.65	0.95	1.09	1.05	0.59	1.37	0.83

C

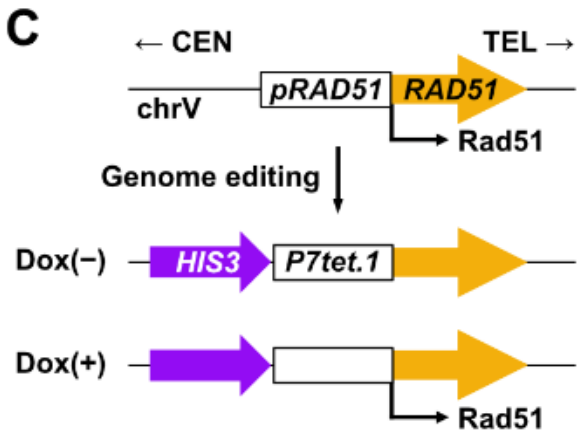

D

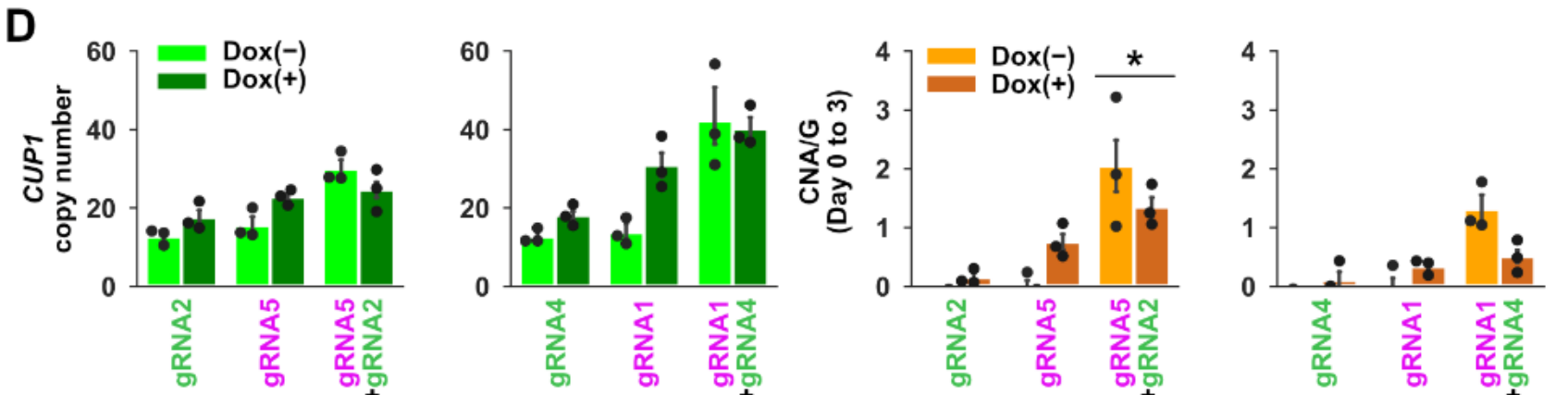

(*legend on next page*)

Figure S3. BITREx 2.0 in mutant cells, related to Figure 4

(A) Growth profiles of mutant strains deficient in canonical BIR and SSA pathways. Generation numbers completed during the 3-day cultivation period are presented. To highlight significant growth defects, gRNA–mutant combinations exhibiting a >2-fold decrease in generation numbers compared to the corresponding gRNA–wild type combinations are heat-mapped in light blue.

(B) Breakdown of fold changes in CNA/G into the contributions of CNA and 1/G. The fold change in CNA/G for each mutant is broken down into the corresponding fold changes in CNA and 1/G. The gRNA–mutant combinations exhibiting >2-fold decreases or increases relative to the corresponding gRNA–wild-type combinations are heat-mapped in light blue and light red, respectively.

(C) Schematic of the Dox-inducible *RAD51* expression. The endogenous *RAD51* promoter is replaced with a Tet-On promoter (*P7tet.1*) for precise, dose-dependent induction of *RAD51* expression upon the addition of Dox (500 ng/mL).

(D) Expansion of *CUP1* array under controlled expression of *RAD51*. Single-nicking BITREx and BITREx 2.0 are induced using the indicated gRNAs in the absence and presence of Dox (500 ng/mL). (Left) *CUP1* copy number at day 3; (Right) CNA/G. Error bar, SD (n = 3 biological replicates). *p < 0.05 (Student's t-test). Note that in single-nicking conditions using gRNA5 or gRNA1, Dox-dependent expansion is observed, consistent with the canonical Rad51-dependent model of BITREx. For BITREx 2.0, however, this inducible system does not fully recapitulate the dramatic hyper-expansion seen in the *rad51*Δ strain. Nevertheless, the depletion of Rad51 (absence of Dox) appears to enhance expansion. The gRNA5+gRNA2 pair shows a significant increase in CNA/G in the absence of Dox. A similar, albeit less pronounced, trend is observed for the gRNA1+gRNA4 combination.

A

Dox	CNA/G			CNA/G		
	gRNA2	gRNA5	gRNA5+2	gRNA4	gRNA1	gRNA1+4
−	0.10	0.06	0.59	0.07	0.26	1.10
+	0.08	0.04	0.39	0.04	0.26	0.37

Dox	$CN_{day\ 3}$			$CN_{day\ 3}$		
	gRNA2	gRNA5	gRNA5+2	gRNA4	gRNA1	gRNA1+4
−	2.04	1.60	7.62	1.78	4.89	14.26
+	2.15	1.53	7.38	1.67	6.65	7.18

B

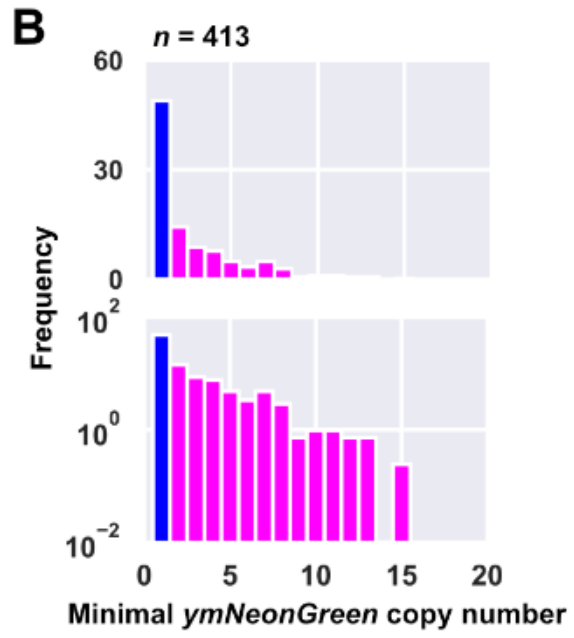

C

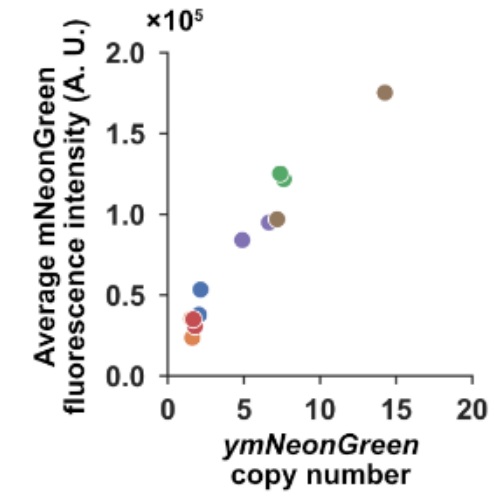

D

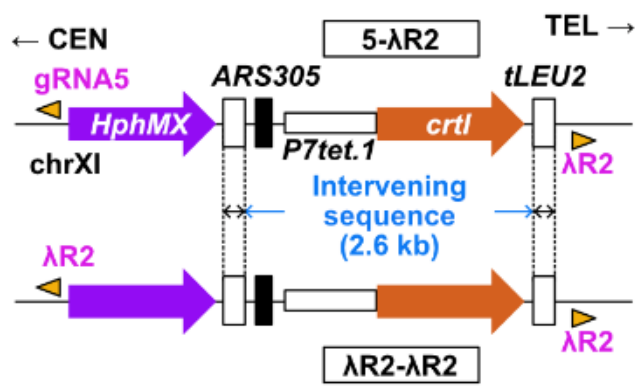

E

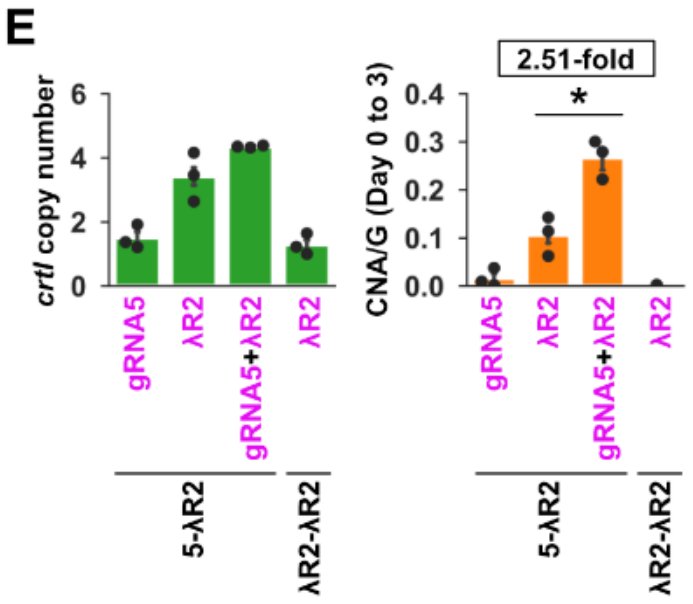

F

Strain	CNA/G		
	gRNA5	gRNAλR2	gRNA5+λR2
5-λR2	0.02	0.11	0.27
λR2-λR2	-	-0.01	-

Strain	$CN_{day\ 3}$		
	gRNA5	gRNAλR2	gRNA5+λR2
5-λR2	1.50	3.42	4.36
λR2-λR2	-	1.29	-

G

ARS(−)

gRNAs		CNA/G			Effect size	Synergy factor	*q*-value (FDR<0.05)
#1	#2	#1	#2	#1+#2			
5	1	0.01	0.15	0.23	0.09	1.52	1.91E-03
2	5	0.03	0.01	0.06	0.04	1.99	5.53E-03
4	1	0.01	0.15	0.43	0.28	2.73	1.03E-06

gRNAs		$CN_{day\ 3}$			Effect size	Synergy factor	*q*-value (FDR<0.05)
#1	#2	#1	#2	#1+#2			
5	1	1.24	5.18	6.59	1.15	1.03	5.10E-02
2	5	1.53	1.24	2.36	0.57	0.85	1.42E-02
4	1	1.26	5.18	10.81	5.35	1.68	2.82E-05

ARS(+)

gRNAs		CNA/G			Effect size	Synergy factor	*q*-value (FDR<0.05)
#1	#2	#1	#2	#1+#2			
5	1	0.01	0.33	0.46	0.14	1.37	1.72E-01
2	5	0.05	0.01	0.17	0.12	2.99	2.93E-02
4	1	0.03	0.33	0.77	0.42	2.13	2.49E-02

gRNAs		$CN_{day\ 3}$			Effect size	Synergy factor	*q*-value (FDR<0.05)
#1	#2	#1	#2	#1+#2			
5	1	1.20	7.55	10.22	2.25	1.17	2.54E-01
2	5	1.24	1.20	2.46	0.81	1.01	1.25E-01
4	1	1.06	7.55	12.20	4.38	1.42	2.24E-01

Figure S4. BITREx 2.0 of interrupted two-unit arrays, related to Figure 5

(A) Summary tables for the data shown in Figure 5B. Two-way ANOVAs were not performed for these strains due to the absence of data for the TEF1 gRNA (negative control).

(B) Distribution of the repeat unit copy numbers within the interrupted *CUP1* array estimated from nanopore sequencing data. Minimal copy numbers are estimated and displayed, as described in Figure S2C. Among the 413 reads containing at least one repeat unit, 211 reads (51.1 %; indicated in magenta) are identified to carry two or more copies.

(C) Correlation between *ymNeonGreen* gene copy numbers and fluorescence intensities. Mean values from Figures 5B and 5C are used for the plot.

(D) Schematic architecture of interrupted two-unit *tLEU2* arrays. The arrays contain an intervening sequence harboring a Dox-inducible *crtI* expression cassette. The upper and lower panels illustrate configurations with asymmetrical target sites (gRNA5 and gRNAλR2) and symmetrical target sites (gRNAλR2 at both flanks), respectively.

(E) Expansion of the interrupted two-unit *tLEU2* array mediated by BITREx 2.0. (Left) *crtI* copy number at day 3. (Right) CNA/G value, with the fold-increase over the expected additive value for the gRNA5+gRNAλR2 combination indicated in the box. Error bar, SD (n = 3 biological replicates). *$p < 0.05$ (Student's *t*-test).

(F) Summary tables for the data shown in (E). Two-way ANOVAs were not performed for these strains due to the absence of data for the TEF1 gRNA (negative control).

(G) Summary tables for the data shown in Figure 5F.

A

CUP1RU number	$CN_{day\ 3}$			Effect size	Synergy factor	*q*-value (FDR<0.05)	$CN_{day\ 3}$			Effect size	Synergy factor	*q*-value (FDR<0.05)
	gRNA2	gRNA5	gRNA5+2				gRNA4	gRNA1	gRNA1+4			
14	21.38	28.83	55.42	22.01	1.10	7.61E-04	18.55	27.36	73.25	44.15	1.60	1.03E-04
2	4.26	4.77	21.42	13.37	2.37	3.67E-02	4.51	7.98	33.69	21.99	2.70	9.15E-03

B

gRNAs		CNA/G			Effect size	Synergy factor	*q*-value (FDR<0.05)
#1	#2	#1	#2	#1+#2			
KRS1F2	RSM10R2	0.03	0.00	0.11	0.07	3.30	8.80E-02
KRS1F4	RSM10R2	0.07	0.00	0.19	0.10	2.35	5.68E-02
KRS1F2	RSM10R4	0.03	0.09	0.23	0.11	1.98	3.48E-03
KRS1F4	RSM10R4	0.07	0.09	0.36	0.19	2.23	5.38E-02

gRNAs		$CN_{day\ 3}$			Effect size	Synergy factor	*q*-value (FDR<0.05)
#1	#2	#1	#2	#1+#2			
KRS1F2	RSM10R2	4.28	3.29	5.64	1.29	0.75	1.85E-01
KRS1F4	RSM10R2	5.42	3.29	7.60	2.12	0.87	1.57E-01
KRS1F2	RSM10R4	4.28	5.99	8.93	1.87	0.87	6.46E-04
KRS1F4	RSM10R4	5.42	5.99	12.07	3.88	1.06	8.88E-02

C

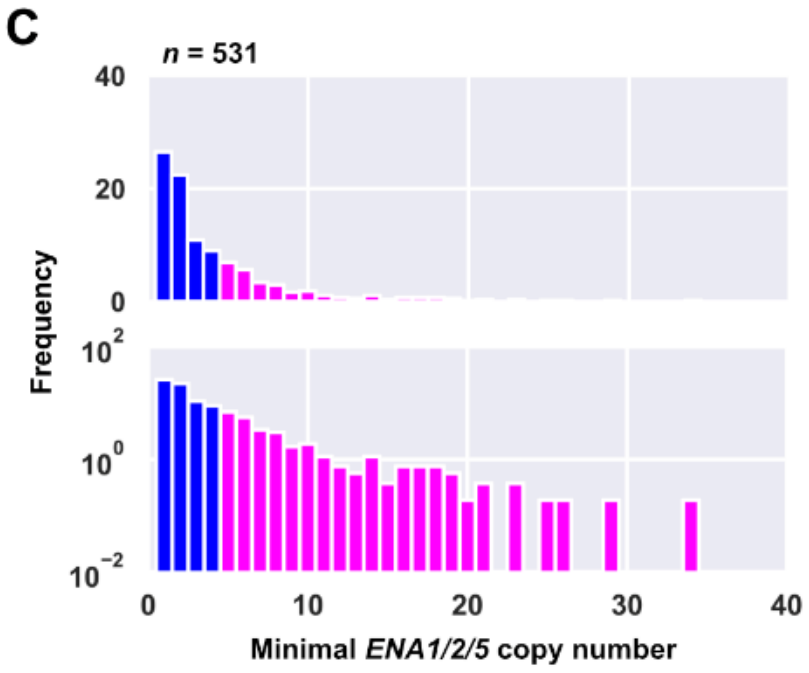

Figure S5. BITREx 2.0 application to expansion-refractory arrays, related to Figure 6

(A) Comparison of BITREx 2.0 efficiency between 14-unit and two-unit *CUP1* arrays. Data are presented similarly to Figure 6C but summarized for absolute CN instead of CNA/G.

(B) Summary tables for the data shown in Figure 6E. CNA/G values below 0.01 are indicated as 0.00.

(C) Distribution of the repeat unit copy number of the *ENA1/2/5* array estimated from nanopore sequencing data. Minimal copy numbers are estimated and displayed, as described in Figure S2C. Among the 531 reads containing at least one *ENA1/2/5* unit, 128 reads (24.1 %; indicated in magenta) are identified to carry six or more copies. While the read with the highest minimal copy-number estimate (34 copies) is truncated within the *ENA1/2/5* array, the read with the second-highest estimate (29 copies) fully spans the entire array; therefore, the latter is used in Figure 6F.
